# Dynamically tunable membrane metasurfaces for infrared spectroscopy and strong light-matter interactions

**DOI:** 10.1038/s41377-026-02382-7

**Published:** 2026-06-09

**Authors:** Furkan Kuruoglu, Samir Rosas, Yihong Chen, Brijesh Kumar, Shenwei Yin, Jin-Woo Cho, David A. Czaplewski, Yuri Kivshar, Mikhail A. Kats, Filiz Yesilkoy

**Affiliations:** 1 Department of Biomedical Engineering, University of Wisconsin-Madison, Madison, WI USA; 2 Department of Physics, Faculty of Science, Istanbul University, Vezneciler, Istanbul, Turkey; 3 Department of Electrical and Computer Engineering, University of Wisconsin-Madison, Madison, WI USA; 4 Nonlinear Physics Center, Research School of Physics, Australian National University, Canberra, ACT Australia; 5 Center for Nanoscale Materials, Argonne National Laboratory, Lemont, IL USA

**Keywords:** Mid-infrared photonics, Optical sensors

## Abstract

Mid-infrared spectroscopy enables biochemical sensing by identifying vibrational molecular fingerprints, but it faces limitations in instrumentation portability and analytical sensitivity. Optical metasurfaces with strong mid-infrared photonic resonances provide an attractive solution towards on-chip spectrometry and sensitive molecular detection, yet their static nature hinders their anticipated impact. Here, we introduce and demonstrate dynamically tunable silicon membrane metasurfaces exhibiting high-Q transmissive resonances in the fingerprint region. By harnessing silicon’s thermo-optical properties, we achieve continuous modulation of coupling-induced transparency (CIT) modes that emerge upon the interference of quasi-bound states in the continuum (q-BICs) and surface lattice modes (SLMs). We measure a spectral tuning rate of 0.06 cm^−^^1^ K^−1^ by continuously sweeping the sharp CIT resonances over a 23.5 cm^−1^ spectral range across a temperature range of 300–700 K. In the current proof‑of‑concept implementation, the dynamic transmission control enables non-contact chemical analysis of polymer films by detecting characteristic absorption bands of polystyrene (1450 and 1492 cm^−1^) and poly(methyl methacrylate) (1730 cm^−1^) without requiring conventional spectrometers. When analyte molecules fill the metasurface-generated photonic cavities, we demonstrate vibrational strong coupling between the poly(methyl methacrylate)’s carbonyl band and the CIT mode, manifested in a Rabi splitting of ~43 cm^−1^. Our results establish a new photonic platform that unites spectral precision, strong field enhancement, and reconfigurability, offering diverse potential for compact mid-infrared spectroscopy, molecular sensing, and programmable polaritonic photonics.

## Abstract (web summary)

Thermally tunable free-standing silicon membrane metasurfaces support high-Q infrared transparency resonances arising from q-BIC and surface lattice mode interactions, offering a compact approach for reconfigurable spectroscopy and biochemical sensing.

## Graphical abstract

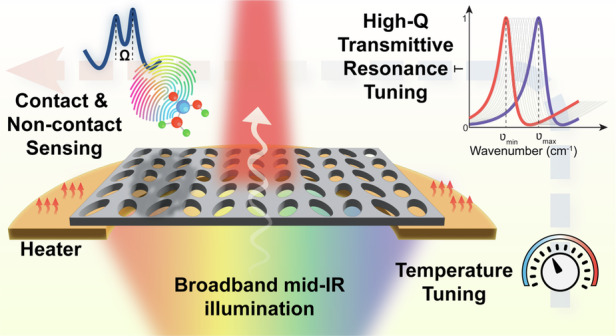

## Introduction

The mid-infrared (mid-IR) spectral range cultivates vibrant photonics research driven primarily by the pressing needs of biochemical sensing and molecular spectroscopy applications. Absorption spectroscopy enables rapid and precise chemical identification by detecting molecules’ distinctive vibrational signatures in the mid-IR region, commonly referred to as fingerprint spectra. Research and development in industrial and academic settings relies heavily on mid-IR spectroscopy using Fourier transform infrared (FTIR) spectrometers and the more recent laser-based discrete frequency interrogation systems. Despite their analytical strength within controlled laboratory settings, these instruments exhibit fundamental constraints, such as large footprints, operational complexity, and high acquisition costs, that obstruct their deployment for widespread, field-based sensing applications. Furthermore, conventional IR spectroscopic techniques typically demonstrate insufficient sensitivity for trace analyte detection due to the inherently limited absorption cross-sections of molecular vibrations (10^−20^ ~ 10^−16^ cm^2^)^1^. These technological and analytical challenges call for innovative photonic approaches to develop field-deployable, highly sensitive mid-IR analytical platforms capable of addressing urgent practical challenges across biomedicine, environmental monitoring, and advanced manufacturing fields.

Optical metasurfaces with engineered subwavelength architectures enable versatile manipulation of IR light, offering transformative solutions for mid-IR absorption spectroscopy^2,3^. To develop compact and spectrometer-less instrumentation, plasmonic mid-IR metasurfaces have been employed as filter arrays to endow microbolometer cameras with spectral selectivity^4–6^. This approach leverages intensity variations in camera pixel readouts to reconstruct spectra or to inform machine learning-based chemical sensing without bulky instrumentation. Other techniques have combined plasmonic perfect absorbers with pyroelectric materials for wavelength-selective IR detectors optimized for compact gas sensors^7^. Beyond their far-field narrow-band filter implementations, metasurfaces have advanced biochemical sensing through their near-field light localization capabilities into subwavelength volumes called hotspots. To date, surface-enhanced infrared spectroscopy (SEIRAS) has enabled important biomedical applications, including living cell^8^, tissue^9^, lipid membrane^10^, and human fluid analysis^11^. Moreover, high quality factor (Q) resonances in dielectric metasurfaces, such as arrays of low-loss Si/Ge structures^12,13^ and perforated free-standing Si membranes^14,15^, significantly enhance light-matter interactions across weak to strong coupling regimes^16^, thereby improving molecular detection^14,15^. However, the static nature of these metasurfaces presents a significant limitation as their high-Q resonances restrict cavity-coupled enhancement to narrow spectral ranges at specific spatial positions, highlighting the need for reconfigurable mid-IR metasurfaces.

Dynamically tunable metasurfaces are highly sought-after components in photonics, because they enable real-time control over resonance frequencies, spectral bandwidths, and wavefront shaping^17–20^. Previously, electrically tunable mid-IR metasurfaces were proposed using GaAs^21,22^, graphene^23–27^ and other van der Waals materials^28,29^ for SEIRAS applications. However, a combination of challenges associated with large-area fabrication of two-dimensional materials and broad resonances created by surface plasmon and phonon polaritons hindered the practical implementations of these devices for chemical sensing. Alternatively, phase-change materials (PCMs), such as Ge₂Sb₂Te₅ (GST)^30–32^, In_3_SbTe_2_^33^ and VO_2_^34^, have been leveraged to endow plasmonic and dielectric metasurfaces with dynamic resonance tunability in the mid-IR. Yet this approach requires incorporating PCMs within the near-field hotspots of resonators to utilize refractive index modulation for active tuning—a configuration fundamentally incompatible with conventional metasurface sensing methodologies. Furthermore, the inherent lossy characteristics of PCMs in the mid-IR region compromise the high-Q resonances of the dielectric resonators. These limitations underscore the critical need for novel photonic approaches to develop reconfigurable high-Q mid-IR metasurfaces optimized for chemical sensing and spectroscopic applications.

Here, we introduce dynamically tunable free-standing Si membrane metasurfaces exhibiting high-Q transmissive resonances in the mid-IR region, functioning as on-demand spectrally selective filter arrays for advanced mid-IR spectroscopy. By harnessing Si’s thermo-optical properties, i.e., its refractive index changes with varying temperatures, we achieve continuous modulation of high-Q (maximum measured 230 @ 1240 cm^−1^) transmission modes in the fingerprint spectral range at a tuning rate of 0.06 cm^−1^ K^−1^. Our metasurface design supports a coupling-induced transparency (CIT) resonance, arising from the interference of broad surface lattice modes (SLMs) and sharp quasi-bound states in the continuum (q-BIC) resonances. Through the temporal modulation of these resonances, we present two sets of experimental demonstrations: i) proof-of-concept spectrometer-free, imaging-based spectral fingerprinting of thin polymer films, poly(methyl methacrylate) (PMMA) and polystyrene, placed in the optical path without direct deposition on the metasurface, ii) vibrational strong coupling between PMMA molecules and dynamically swept metasurface resonances as they traverse the molecule’s fundamental vibrational mode. Our results show that real-time tunability of sharp mid-IR resonances enables chemical analysis of objects positioned in the optical path, without direct physical contact to the metasurface. Moreover, strong near-field light localization within accessible air voids leads to quantum coherent light-matter interactions, with profound implications for ultrasensitive molecular detection. With promising advancements towards compact analytical instrumentation and enhanced sensing capabilities of the mid-IR spectroscopy, our approach offers a potential pathway toward addressing the analytical needs of biomedicine, industrial manufacturing, and environmental safety.

## Results

**Fig. 1:**
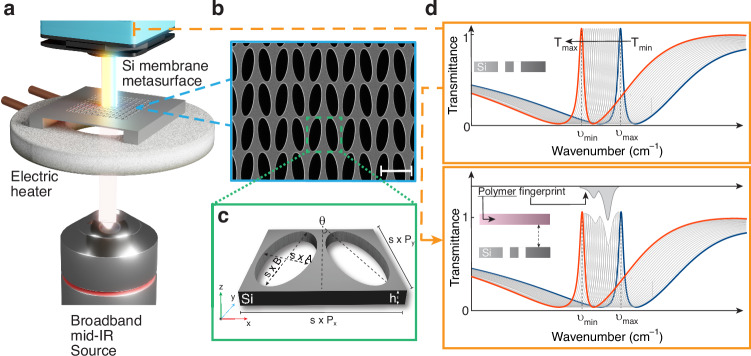
Dynamically tunable Si membrane metasurfaces for mid-IR spectroscopy. **a** Schematic of the optical characterization system in transmission mode, where a mid-IR source illuminates the metasurface placed on an electrically controlled heating stage and transmission spectra are collected as the temperature of the stage is varied, **b** Top-view SEM image of the fabricated Si membrane metasurface, illustrating the periodic perforations that form its lattice (scale bar = 5 μm), **c** Three-dimensional schematic of the meta-unit, highlighting the key geometric parameters, including the periodicities (*P*_*x*_*, P*_*y*_), membrane thickness (*h*), elliptical tilted rod apertures with major and minor axes (*A, B*), tilting angle (*θ*) and geometric scaling factor (*s*), **d** Temperature-dependent transmission spectra of the metasurface as a function of wavenumber (${\boldsymbol{\nu }}$). A continuous redshift in the resonance position is observed at a tuning rate of 0.06 cm^−1^ K^−1^, due to the temperature-dependent refractive index variation of the Si membranes. The dynamic resonance tunability enables the measurement of the characteristic fingerprints of the analytes, whether spatially separated from the metasurface in a non-contact configuration or directly interfaced for enhanced near-field interactions

To investigate the thermal tunability of the Si membrane metasurfaces, we first performed temperature-dependent optical characterization using a broadband mid-IR source integrated with the FTIR microscope and an electrically controlled heating stage, as illustrated in Fig. 1a. Our photonic device supports a sharp transmissive resonance, wherein a broad multipolar SLM interferes with a narrow q-BIC mode, achieved by oppositely tilting an aperture rod pair in a meta-unit (Fig. 1c). The arrayed tilted rods were patterned through 1 μm thick single-crystalline Si membranes by electron beam lithography and anisotropic dry etching, as detailed in the Methods section (Fig. 1b). Upon thermal stimulation, the intrinsic thermo-optical properties of crystalline Si facilitate controlled modulation of its complex refractive index^35,36^, introducing a pronounced spectral shift of the resonance peak, as depicted in Fig. 1d. By spectrally sweeping the transmissive resonance peaks across the fingerprint spectra, we detected the characteristic absorption bands of analytes, whether spatially separated from the metasurface in a non-contact configuration or directly interfaced for enhanced near-field interactions.

**Fig. 2:**
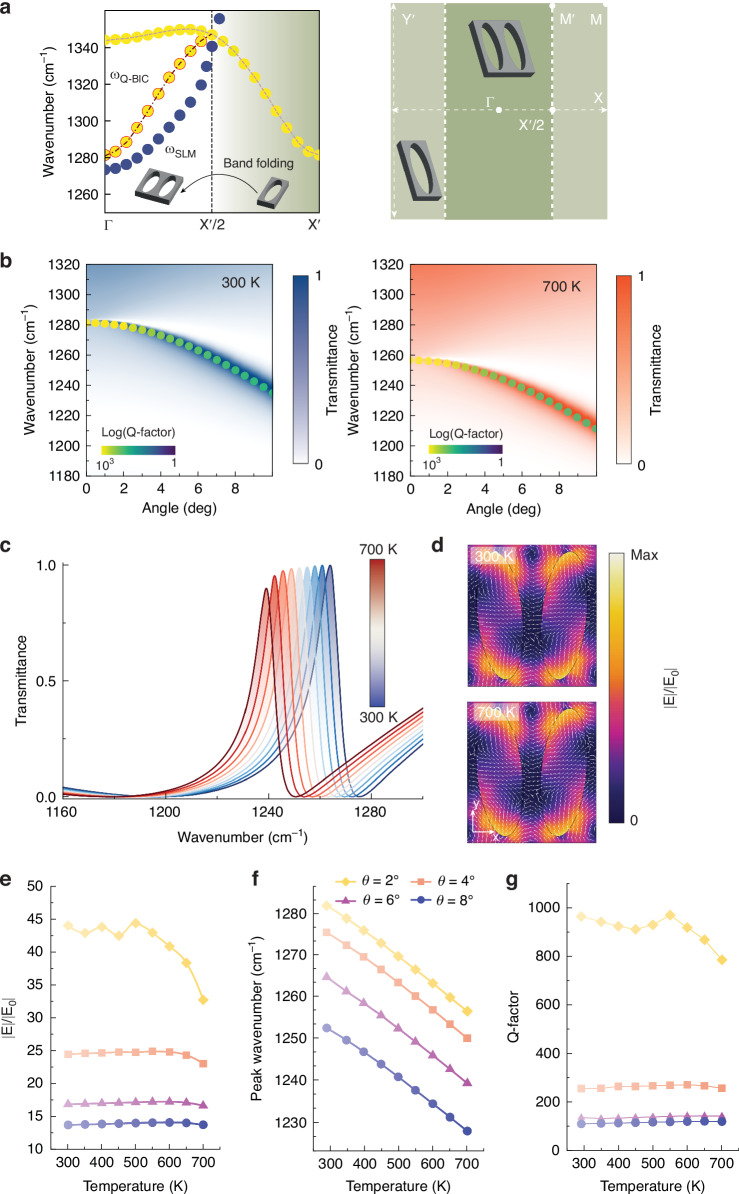
Thermal tuning of q-BIC-CIT resonant modes-simulation results. **a** Brillouin-zone folding resulting from expansion of the unit cell from monoatomic to diatomic along the x-direction brings a mode from the X point to Γ, where it forms a bound state in the continuum in close proximity to the surface lattice mode. Eigenfrequency dispersion along Γ–X illustrates the folded mode, which evolves into a q-BIC under antisymmetric tilting of the elliptical apertures. Insets show the corresponding unit-cell geometries. On the right panel, Γ, X, Y, and M denote high-symmetry points of the reciprocal lattice (Brillouin zone) and are shown for schematic reference only. **b** The transmittance colormaps and the associated Q-factors show the overall effects of the temperature on the spectral shift and of the q-BIC-CIT modes supported by the Si membrane metasurfaces. **c** Numerically simulated transmittance spectra of the Si membrane metasurface with *θ* = 6° tilted elliptical rods supporting photonic analog of CIT resonances. Spectral resonance tuning was achieved by varying the metasurface temperature from 300 K to 700 K. **d** The electric (E)-field enhancement maps and displacement current density arrow profiles on a meta-unit surface show that the E-field enhancement profile remains the same for different temperatures. **e** The maximum E-field enhancement as a function of temperature. |E | / | E_0_| degrades at higher temperatures for all tilting rod angles, with a higher gradient at smaller tilting angles. **f** The resonance peak wavenumber shifts as a function of temperature, showing thermal tuning rates for each rod tilting angle. **g** The Q-factor of the resonance as a function of temperature

To investigate the physical origins of the narrowband transmissive resonance, we employed finite-element simulations and eigenmode analysis. We first calculated the eigenfrequencies of a periodic array of single-elliptical-holes (monoatomic unit cell) along the $\varGamma -X$ direction, in which a broad SLM and a high-Q guided mode coexist. Expanding the unit cell along x-direction to include two elliptical holes (diatomic unit cell) folds the Brillouin zone from Γ→X to Γ→X/2. The Brillouin zone folding (BZF) introduces a BIC mode near the SLM at the Γ-point, which was originally located at X in the monoatomic unit cell (see details in SI I.A). A schematic comparison of the Brillouin zones for the monoatomic and diatomic unit cells and the corresponding eigenfrequency dispersions are shown in Fig. 2a.

In our metasurface design, the unit cell becomes diatomic, and BZF occurs intrinsically when neighboring elliptical apertures are oppositely tilted. Moreover, the in-plane symmetry breaking converts the BZF-BIC into a q-BIC mode, enabling far-field access. At the design phase, we also investigated alternative perturbation schemes that can potentially support BZF-induced q-BIC modes in membrane metasurfaces; however, none of these approaches were as effective as our rod tilting approach (see details in SI I.B and Fig. S2).

Contrary to previously reported q-BIC modes^12–14^, our metasurface design supports a transmissive resonance. We investigated this unique photonic phenomenon using numerical simulations and the temporal coupled mode theory as detailed in SI I.C. and SI II. We first identified that the high-Q transmissive mode is a coupling-induced transparency (CIT) because it emerges when the q-BIC mode couples to the SLM mode upon spectral and spatial overlap. To further investigate the effect of spectral proximity between the SLM and the q-BIC on the CIT, we varied the unit cell period (*P*_*x*_), which spectrally shifts SLM and q-BIC modes at different rates (see details in SI I.C. and Fig. S3). Our results showed that the q-BIC-CIT mode gets distorted as the SLM and q-BIC modes spectrally separate, indicating the mode coupling effect. Moreover, when the simulated spectra were fitted to the CIT model derived based on the temporal coupled mode theory, we observed a strong agreement (see details in SI II.D. and Fig. S4).

To analyze the effects of thermal tuning on the q-BIC-CIT mode, we first performed numerical simulations using the temperature-dependent complex refractive index of Si^35–37^. Figure 2b shows transmittance maps as a function of rod tilting angle (*θ* = 0°–10°) at 300 K and 700 K. The filled circles in the plots mark the eigenmodes of the q-BIC, which align well with the CIT peak positions. The filling color of the circles indicates the Q-factor of the mode, which decreases with increasing tilt angle, $\theta$, at both temperatures. The q-BIC-CIT mode properties are highly dependent on the geometric design parameters, where the tilt angle, $\theta$, primarily controls radiative leakage, and thus the resonance amplitude and Q-factor, and the symmetry-preserving parameters (membrane thickness *h*, lattice periodicities *P*_*x*_*, P*_*y*_, and aperture dimensions *a, b*) tune the resonance wavelength and the mode coupling (see details in SI IV and Fig. S6).

Figure 2c shows simulated transmission spectra as the temperature varies from 300 K (room temperature, RT) to 700 K in 50 K increments for tilt angle *θ* = 6°. A total spectral shift of 25.6 cm^−1^, greater than the full width at half maximum (FWHM) of the resonance (11.4 cm^−1^), was achieved with a temperature difference of 400 K. In the elevated temperature range, the transmittance of the membrane metasurface exhibited a reduction (9% at 700 K compared to RT) due to the increased material losses. To elucidate the impact of temperature on the q-BIC-CIT resonance characteristics of the membrane metasurfaces, we plotted the electric (E) field enhancement and displacement current profiles for the minimum (300 K) and maximum (700 K) measured temperatures at the resonance. Figure 2d shows that the E-field enhancement (*E*_*enh*_ = *|E* | */* | *E*_*0*_ | ) maintains a consistent profile at varying temperatures, with hotspots emerging at the tips of the elliptical apertures. The robustness of the q-BIC-CIT mechanism at elevated temperatures is further confirmed by the consistent phase response of the mode observed at varying temperatures (details in SI V and Fig.S7).

To further quantify the temperature-dependent q-BIC-CIT mode properties, we plotted the E-field enhancement, peak wavenumber, and Q-factor as a function of temperature for different rod tilting angles (*θ* = 2°, 4°, 6°, and 8°) in Fig. 2e, f, and g, respectively. The *E*_*enh*_ and Q-factor are higher for smaller rod tilting angles across the entire temperature range, a characteristic of q-BIC modes^38–40^. At high temperatures (T > 550 K), *E*_*enh*_ *and Q-factor* decrease consistently for all *θ* values, with a higher gradient for smaller *θ*. We provide a detailed quantitative analysis of temperature-dependent E-field enhancement in SI VII and Table S2. Moreover, to investigate the relative contributions of radiative and non-radiative damping to Q-factor reduction, we decomposed the temperature-dependent loss channels (see SI VI and Fig. S8). The results indicate that the reduction of the Q-factor at elevated temperatures is dominated by an increase in non-radiative damping, while the radiative loss channel remains comparatively insensitive to temperature and primarily controlled by the rod tilting angle. In addition, silicon oxidation in this temperature regime remains self-limiting and induces only a negligible resonance shift without measurably affecting the Q-factor (see SI VIII and Fig. S9).

As the temperature increases, a clear redshift in the resonance position is observed for all metasurface designs (Fig. 2f). The highest thermal resonance tunability rate was calculated to be 0.064 cm^−1^ K^−1^, for a rod tilting angle of *θ* = 2°.

**Fig. 3:**
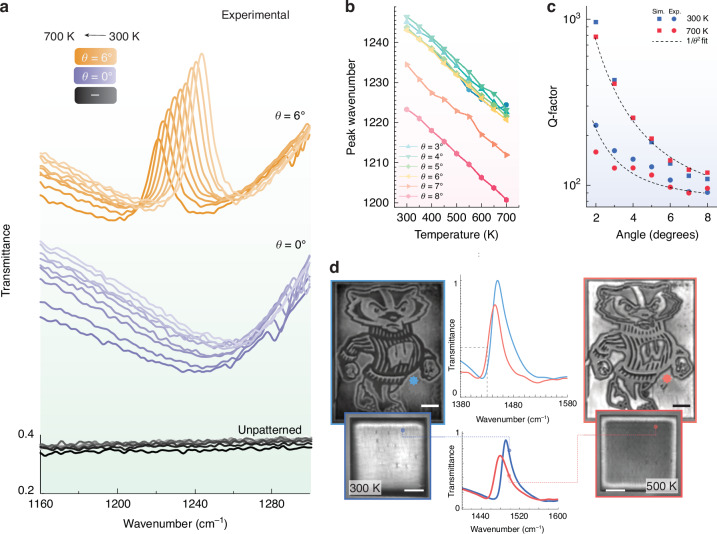
Experimentally measured thermal tuning effects on the photonic q-BIC-CIT modes. **a** Temperature tunability characteristics of the Si membrane metasurfaces with *θ* = 6° (yellow lines) showing q-BIC-CIT mode and *θ* = 0° (violet lines) showing SLM, where q-BIC vanishes to BIC, both red shifting as the real part of the refractive index increases with increasing temperature. Black lines show transmission through the unpatterned membrane. **b** Measured resonance peak wavenumber as a function of temperature for different tilted rod angles. For all angles, the measured thermal tuning rate is *Δv/ΔT* ≈−0.05 cm^−1^ K^-1^. **c** Evolution of Q-factor as a function of rod tilting angle *θ* for simulation and experimental data, showing inverse quadratic correlation (Q-factor∝ *θ*^−2^) for different temperatures. **d** Wide-field IR microscope images of the UW-Madison mascot, Bucky Badger, patterned metasurface, measured by illuminating at 1430 cm^−1^ at 300 K and 500 K temperatures (Scale bar = 250 μm). As the temperature increases, the resonance peak shifts to lower wavenumbers, leading to a contrast inversion, as also indicated on the FTIR measured transmittance spectra. A similar contrast inversion effect is shown on a different uniformly patterned metasurface, where the bright to dark transition occurs at 1498 cm^−1^ as temperature increases (Scale bar = 100 μm)

Next, we measured the temperature-dependent resonance characteristics of fabricated Si membrane metasurfaces with varying rod tilting angles and compared them with the simulation results. Figure 3a shows the mid-IR transmittance spectra obtained from the unpatterned Si membranes for reference and fabricated metasurfaces with rod tilting angles *θ* = 6° and 0°. At *θ* = 6°, a temperature-tuned q-BIC-CIT mode is observed; however, at *θ* = 0° only the SLM is present as the q-BIC vanishes to BIC and the transparency window disappears. Figure 3b illustrates that all measured metasurfaces with varying rod tilting angles exhibit a consistent linear shift in their resonances across the examined temperature range (300–700 K). In strong agreement with the simulation results, the high-Q metasurface (*θ* = 2°) demonstrates the highest tunability rate at 0.06 cm^−1^ K^−1^, while the tunability rate of the SLM (*θ* = 0°) is 0.051 cm^−1^ K^−1^. We also tested the robustness of the temperature-dependent resonance response, finding that the temperature-induced resonance shift is identical in the heating and cooling cycles, and the spectral response does not significantly degrade after repeated (*n* = 20) thermal cycles (see details in SI IX and Fig. S10).

Figure 3c shows the experimentally derived Q-factors from metasurfaces with varying rod tilting angles at the lowest (300 K) and highest (700 K) measured temperatures. The inverse quadratic relationship of the Q-factor with the asymmetry parameter (*θ*) indicates the q-BIC nature of the measured resonances at both temperatures^41^. Notably, at elevated temperatures (T > 550 K), we observed a more pronounced reduction in mode amplitudes and Q-factors in experimental spectra when compared with the simulation results. Our further investigation revealed that the standard simulation workflow fails to capture the resonantly enhanced light–matter interactions inside the silicon slab. Therefore, as the membrane temperature increases, absorption pathways in c-Si become more pronounced due to the resonant metasurface. Thus, the effective absorption scales with the local field intensity, as we discuss and demonstrate in SI X and Fig. S11.

To visualize the dynamic resonance tunability characteristics, in Fig. 3d, we show the mid-IR microscope images of a partially patterned metasurface in the shape of Bucky Badger, UW-Madison’s mascot. The Bucky metasurface images were captured at 1430 cm^−1^ illumination in transmission mode at two different temperatures (300 K and 500 K). Due to a dynamic redshift in the resonance peak upon temperature increase, metasurface patterned regions become more transmissive at the same illumination wavenumber (1430 cm^−1^) revealing a dark-to-bright contrast transition. This effect is also depicted in the transmittance spectra measured at the indicated red and blue star positions of the images at 300 K and 500 K. With only a 200 K temperature increase, a notable transmittance boost from 0.23 to 0.39 (68.2%) was measured. We also show an opposite bright-to-dark transition by probing the resonance peak of a uniformly patterned metasurface area of 400 µm × 400 µm at 1498 cm^−1^, which exhibits a 58.7% decrease in transmittance as a result of a 200 K temperature increase (illustrated in the lower segment of Fig. 3d).

**Fig. 4:**
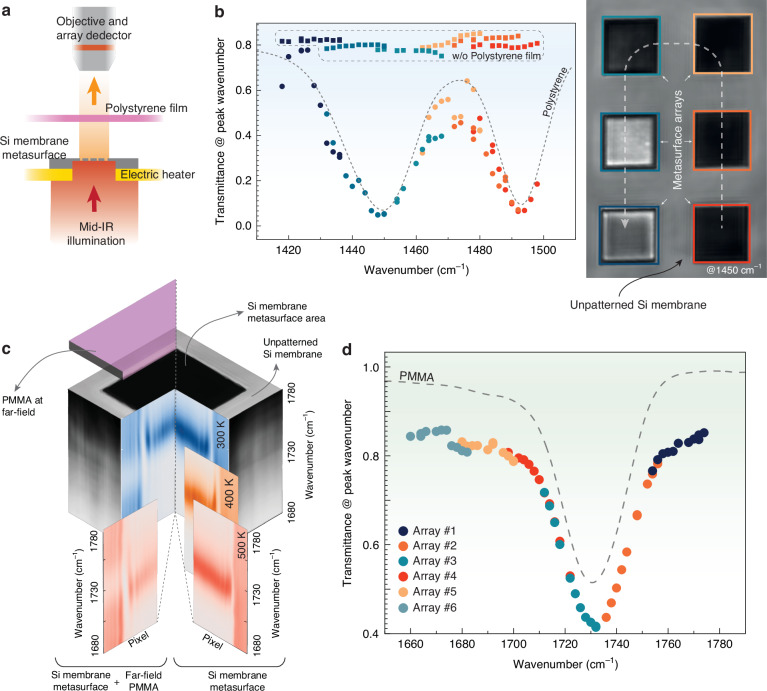
Non-contact mode chemical analysis of polymer films positioned in the optical path using dynamically tunable metasurfaces. **a** Schematic illustration of the material analysis setup where a polymer film is placed at least 10 mm distant from the thermally tuned metasurface. **b** The maximum transmittance of six different metasurfaces was measured at varying temperatures from 300 to 500 K, with and without a polystyrene film (thickness = 38 μm). Each metasurface covers around 18 cm^−1^ spectral range upon a 200 K temperature sweep. Six metasurfaces were sufficient to capture absorption fingerprints of the polystyrene including modes at 1450 and 1492 cm^−1^ corresponding to C=C stretching vibrations. The colors of the data points correspond to the colored frames of the metasurfaces in the IR microscope image captured at 1450 cm^−1^. **c** The hyperspectral data visualization of a single metasurface showing temperature-dependent spectral shifts at three discrete temperatures, 300 K, 400 K, and 500 K. The left panel shows measured data with PMMA film, whereas the right panel depicts the bare metasurface. Spectral colormaps effectively demonstrate spectral homogeneity across the metasurface area, both in the presence and absence of the PMMA film. Corresponding to the PMMA C=O absorption band at 1730 cm^−1^, the resonance transmission of the metasurface decreases as it is thermally swept through the band. **d** The maximum transmittance of six metasurfaces, each with a different resonance wavenumber, are measured at varying temperatures from 300 to 500 K, with and without a PMMA film of 700 nm thickness. In (**b**) and (**d**), the gray dashed curves correspond to independently measured reference absorption spectra of the polystyrene and PMMA films, respectively

We leveraged the temperature tunability of the Si membrane metasurfaces as dynamic narrow-band mid-IR filters to demonstrate non-contact chemical analysis of polymer films. Figure 4a depicts the transmission-mode optical imaging configuration, wherein the spectral responses of an array of metasurfaces were characterized while modulating the substrate temperature. An example of this chemical analysis is shown in Fig. 4b, where we employed an array of six metasurfaces with predetermined resonance wavenumbers assigned during fabrication. When a polymer film specimen is inserted into the optical path, the characteristic vibrational absorption signatures of the molecular constituents induce distinct transmission intensity variations across the metasurface array elements, each exhibiting different magnitudes of attenuation as their resonance frequencies are thermally tuned. In Fig. 4b, the frame color of each metasurface in the mid-IR image corresponds to the color of the spectral data points presented in the accompanying plot. Square and circular data points represent the transmittance values of each metasurface at varying temperatures in the absence and presence of a polystyrene film, respectively. By continuously sweeping the metasurface array temperature from 300 to 500 K, we acquired a continuous fingerprint spectrum of polystyrene, clearly resolving the 1450 cm^−1^ and 1492 cm^−1^ absorption bands associated with (C=C stretching) benzene ring vibrations, respectively. Similarly, we demonstrated detection of a thin PMMA film, resolving its characteristic carbonyl (C=O) stretching vibration at 1730 cm^−1^ (Fig. 4d). For both polymer species, our approach accurately determined the spectral position and FWHM of the absorption bands, with measured transmission modulation aligning closely with reference spectra obtained through conventional spectroscopy (gray dashed lines in Fig. 4b and d).

To characterize the spatial resonance characteristics of our thermally tunable metasurfaces, we used a hyperspectral imaging method. Figure 4c presents spectral cross-sections extracted from a hyperspectral image datacube of a single metasurface (area: 400 × 400 μm^2^) acquired with and without a PMMA film at discrete temperatures of 300, 400, and 500 K. Spatially resolved transmittance profiles within the metasurface element show the shift in resonance peak toward 1730 cm^−1^ upon temperature increase from 300 to 500 K. The spectral overlap of the metasurface resonance and the characteristic carbonyl absorption band of PMMA at 1730 cm^−1^ induces a transmission attenuation across the entire metasurface.

While Fig. 4c shows a single metasurface for clarity in data presentation, our optical system uses a mid-IR objective (0.3 NA) and a focal-plane detector, imaging a field of view of approximately 2 mm × 2 mm. Therefore, our imaging optics can integrate a large array of metasurfaces (e.g., 36 of area 200 µm × 200 µm) allowing parallel interrogation across a wider spectral range. Given that a single metasurface can be tuned across a ~ 23 cm^−1^ spectral range when heated from 300 K to 700 K, a 36-element metasurface array can cover a bandwidth of ~ 820 cm^−1^, providing multi-band monitoring across the fingerprint spectra in a single frame without mechanical scanning.

To ensure uniform temperature distribution and homogeneous fabrication process, we investigated spectral response uniformity both within a single metasurface and across an array of metasurfaces distributed over a large membrane area (~3 mm × 3 mm) (see SI XI and Fig. S12). Considering the expected fabrication imperfections and intrinsic mode degradation towards the metasurface edges, the spatial maps of resonance wavenumbers at 300 K and 500 K exhibit spatial uniformity, with standard deviations of σ ≈ 8.00 cm^−1^ and σ ≈ 10.55 cm^−1^, respectively. Moreover, the thermally induced resonance shift across the membrane remains comparable to the minimum resolvable spectral step (2 cm^−1^) of our optical system, indicating uniform temperature distribution across the membrane.

Next, we investigated the transient thermal response of the free-standing Si membranes to provide insight into the dynamic tuning speed of our photonic system. Owing to low thermal mass and high in-plane thermal conductivity of the membrane, thermal equilibration occurs at millisecond timescales, with smaller area membranes reaching the steady state thermal equilibrium faster than the larger ones, as shown by the numerical simulations (details in SI XII and Fig. S13).

**Fig. 5:**
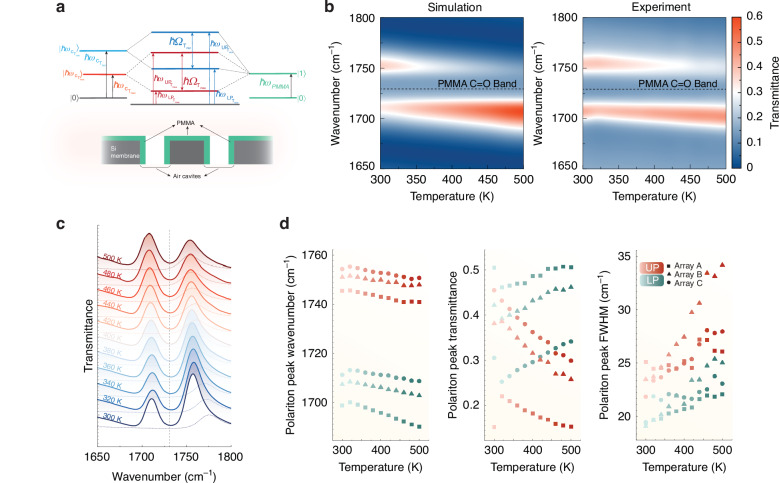
Near-field vibrational strong coupling realized with dynamically tunable metasurfaces. **a** Upon coating the free-standing Si membrane metasurface with 50 nm thick PMMA, near-field light-matter interactions were studied. When the low-loss q-BIC-CIT mode spectrally overlaps with the PMMA’s absorption band, the vibrational strong coupling condition is satisfied, and hybrid light-matter states (polaritons) are generated. The schematic shows the upper (UP) and lower (LP) polariton states separated by the Rabi splitting (Ω) at two different resonance frequencies (blue and red lines) of the same metasurface achieved by temperature tuning. **b** Simulated and experimentally measured transmittance spectra of a PMMA-coated metasurface at varying temperatures from 300 to 500 K. The temperature-dependent transmittance maps show the Rabi splitting between the upper and lower polariton branches. The pronounced anti-crossing behavior captured by a single dynamically tunable metasurface confirms vibrational strong coupling over the entire temperature range. **c** Measured FTIR spectra demonstrating the transmittance variation of LP and UP as the metasurface resonance is swept through the PMMA vibrational band. **d** The upper (red) and lower (green) polariton peak wavenumber, maximum transmittance, and FWHM variations as a function of temperature measured from three different metasurfaces

To investigate the near-field light-matter interactions on the thermally modulated metasurfaces, we deposited PMMA thin films onto Si membrane metasurfaces. Leveraging the low material dissipation and high-Q resonances of the Si membrane metasurfaces, we previously demonstrated vibrational strong coupling (VSC) between the PMMA’s carbonyl (C=O) band and q-BIC and q-BIC-CIT resonance modes^14,15^. Here, we study coherent energy exchange dynamics and formation of hybrid light-matter states (polaritons) while dynamically sweeping the metasurface resonance mode through PMMA’s vibrational band. Figure 5a illustrates the hybrid polaritonic energy states, depicting lower polariton (LP) and upper polariton (UP), separated by the Rabi frequency (Ω) associated with VSC at two distinct metasurface resonance levels. Figure 5b presents the simulated and measured spectral maps showing VSC-associated anti-crossing behavior as the metasurface temperature gradually increases and q-BIC-CIT mode traverses the PMMA band. The measured transmittance spectra across a 200 K temperature gradient in Fig. 5c demonstrate that as the temperature increases and the q-BIC-CIT mode shifts to lower wavenumbers, the intensity of the UP gradually diminishes, while LP rises. We comprehensively characterized the polariton parameters in Fig. 5d, where temperature-dependent UP and LP peak wavenumber, transmittance amplitude, and linewidth data are presented. As the metasurface temperature increases and its resonance shifts to lower wavenumbers, LP couples more efficiently to the free space propagation than the UP, resulting in an enhanced LP transmission signal. Furthermore, we observed a systematic increase in polariton linewidths with temperature. Temperature-dependent measurements of the PMMA reference film between 300 and 500 K reveal negligible variation in the intrinsic vibrational FWHM of the C=O band (see SI XIII and Fig. S14). This suggests that the temperature-induced polariton linewidth broadening mainly results from increased material loss, phonon interactions, and scattering processes at elevated temperatures, contributing to a reduction in coherence lifetime of the hybrid states.

## Discussion

Our work introduces the thermally tunable free-standing Si membrane metasurfaces exhibiting high-Q transmissive resonances in the mid-IR molecular fingerprint region. By leveraging Si’s thermo-optical properties, we achieved temporal control over metasurface resonance characteristics. Specifically, our imaging-based optical interrogation allows for the simultaneous interrogation of arrays of metasurfaces over large areas, each covering a distinct spectral range via dynamic temperature modulation. Thus, our approach enables chemical analysis of non-contact objects by identifying their absorption bands across the fingerprint spectrum. Furthermore, recent successful integration of plasmonic metasurfaces with compact illumination and detector architectures^4,6,42^ support the feasibility of miniaturizing our technology for on-site and low-cost chemical sensing applications.

Previous strategies to achieve broadband mid-IR tunability in high-Q dielectric metasurfaces often required mechanical components or spatially encoded designs. For instance, Ge metasurfaces were optically interrogated by sweeping the illumination angle to modulate the spectral positions of their q-BIC resonances. However, this approach depends on goniometric setups and moving parts, which limit their robust integration^13^. More recently, gradient metasurfaces have enabled broadband functionality by spatially encoding resonance frequencies over large areas via gradual variations in unit cell geometry^43,44^. However, to keep the gradient metasurface footprints small, it is necessary to rapidly change resonator geometric parameters across short distances, which modifies local radiative coupling conditions and reduces the Q-factors. Thus, the gradient approach usually trades spectral bandwidth for resonance strength. In contrast, our device uses arrays of uniform metasurfaces, which preserve the high-Q resonances across the entire field of view. Notably, our thermal tuning approach can be applied to gradient metasurfaces to further expand their bandwidth or shift resonance distributions on demand across the gradient. Therefore, static gradient and dynamic thermal tuning approaches are complementary rather than competitive methods.

Our transient thermal simulations indicate that our current bulk thermal tuning approach using an external ceramic heater yields tuning speeds in the millisecond range. While this temporal response is sufficient for our chemical sensing applications, it can be expedited using electrode-based localized thermal control schemes for photonic applications requiring faster response. Importantly, we did not observe any deformation, bowing, or buckling on the membranes at any stage of the heating process (see Fig. S15), confirming that the suspended Si membranes are mechanically robust to rapid thermo-optical tuning within the investigated temperature range, which is practically bounded by the temperature dependence of Si’s thermo-optic coefficient. Below 300 K, the reduced dn/dT yields a smaller resonance shift and limited tuning efficiency, while temperatures above ~700 K provide no additional spectral benefit.

Our experimental results showed a significant Q-factor degradation at metasurfaces with small rod tilting angles (*θ* = 2°) at temperatures above 550 K. This is not a major hindrance for our chemical sensing applications because in our experimental analysis, we employed metasurfaces with *θ* = 6° as a balanced operating point, which is quantitatively supported by a figure of merit defined as ${FoM}=Q\times {I}_{{peak}}$ (see SI XV and Fig. S16). The modest linewidth changes in these metasurfaces at elevated temperatures do not hinder chemical identification, since mid-IR vibrational absorption bands are intrinsically broad. Thus, our approach clearly identified the characteristic absorption bands of polystyrene (1450 and 1492 cm^−1^) and PMMA (1730 cm^−1^) when the samples were positioned several millimeters above the metasurface.

This work represents a step towards compact analytical tools demonstrating spectrometer-free chemical fingerprinting. In practice, the metasurface can be combined with a broadband mid-IR source and an IR camera in a simple transmission-mode wide-field imaging setup, leveraging emerging high-performance mid-IR detector technologies^45–47^. During on-site chemical sensing, the analyte’s fingerprint spectrum can be retrieved from a series of intensity-only measurements using a priori recorded temperature-dependent spectral response of the metasurface, eliminating the need for dispersive or interferometric optics (see SI XVI and Fig. S17). In the current implementation, the dynamic tuning bandwidth is limited by the thermo-optic coefficient of Si. Further expansion of the spectral bandwidth would require integration of materials with higher thermo-optic coefficients, such as Ge and III–V semiconductors^48^.

While our method relies on high-temperature operation, the heated area is confined to the suspended membrane, which is distant from the sample to be analyzed. For heat-sensitive analytes or those with weak vibrational signals, thermal effects can hinder reliable measurements. Therefore, our present operating conditions are primarily suited to thermally robust analytes. To suppress the adverse thermal effects, localized membrane heating can be used, to minimize the heated mass, or the sample can be thermally isolated from the metasurface by inserting an IR-transparent window, such as CaF_2_. Finally, active cooling can be integrated when the application requires high sensitivity, at the expense of instrumentation complexity and cost.

In the near-field, our photonic device enables exceptional light-matter interaction capabilities in the strong coupling regime. Previously, vibrational strong coupling was studied in Fabry-Perot cavities, sweeping their resonances by angular tuning^49^, or on low-loss dielectric metasurfaces by spatially varying resonances^14,15^. Our dynamically reconfigurable metasurfaces generate hybrid light-matter states, polaritons, where thermal and frequency variations act together to define the coupling dynamics and coherence properties. Such real-time characterization capabilities introduce new experimental means to further investigate the fundamental physics of hybrid light-matter states, without the need to sequentially measure spectra by varying spatial position or illumination angle.

In conclusion, we have demonstrated that our dynamically reconfigurable Si membrane metasurfaces provide a versatile platform for advanced mid-IR spectroscopy and sensing. The combination of high-Q transmissive resonances, dynamic tunability, and accessible photonic cavities makes our approach well-suited for integration into compact, sensitive, and field-deployable chemical analyzers. By addressing the key limitations of mechanically tuned and spatially distributed metasurfaces, this work opens new opportunities in reconfigurable IR spectroscopy, vibrational polaritonics, and many other applications of actively tunable light–matter interaction systems. While this study represents a proof-of-concept demonstration, the proposed architecture establishes a practical route toward fully integrated, field-deployable analytical systems.

## Methods

### Numerical simulations

The finite element method simulations were performed using COMSOL Multiphysics (COMSOL AB, Sweden) to calculate the transmittance spectrum, electric field enhancements, and displacement current density in Figs. 2 and 5. In all simulations, Floquet periodic boundary conditions were applied along the x- and y-axes, while the top and bottom boundaries in the z-direction were modeled as perfectly matched layers (PML). To excite the q-BIC-CIT resonances, an x-polarized plane wave was used at normal incidence. We used the temperature-dependent refractive index of silicon^35,37^ to simulate the spectral response of the metasurface.

### Fabrication

The dynamically tunable membrane metasurfaces were fabricated using conventional semiconductor process steps. We first fabricated intrinsic single-crystal Si membranes measuring approximately 1 µm in thickness and 2.8 mm × 2.8 mm in area, supported by a 300µm-thick Si frame with dimensions of 10 mm × 10 mm using photolithography and wet/dry Si etching processes. The metasurfaces were fabricated by patterning the free-standing Si membranes using electron beam lithography (EBL) for mask formation, followed by reactive ion etching (RIE) to anisotropically etch the Si and generate tilted rod voids with vertical, smooth sidewalls. Further details of the fabrication process can be found in Rosas et al.^15^.

### Optical characterization

Mid-infrared characterizations of the metasurfaces were performed in transmission mode using collimated, linearly polarized light at normal incidence on two different platforms: an FTIR system with a cryostat stage and a discrete frequency IR (DFIR) microscope with a ceramic electric heater. Figure 3 was generated using data collected by the FTIR spectrometer, coupled to an infrared microscope (Bruker Vertex 70 FTIR and Hyperion 2000). Transmittance spectra were acquired using linearly polarized light focused by a low-NA refractive objective (5×, 0.17 NA, Pike Technology, WI, USA). Collimated illumination was achieved by removing the bottom condenser, and detection was carried out using a liquid-nitrogen-cooled mercury-cadmium-telluride (MCT) detector. For temperature-dependent measurements, the metasurface was mounted directly on a cryostat stage (Linkam Scientific, FTIR600), and the temperature was swept from 300 K to 700 K.

For imaging-based spectral interrogation, we used a DFIR system integrated with four tunable quantum cascade lasers (QCL) on a microscope setup (Daylight Solutions, CA, USA), as shown in Figs. 3–5. The QCL microscope recorded the mid-IR spectrum in the range of 950 to 1800 cm^−1^ with a spectral resolution of 2 cm^−1^. Spectral data were collected using a 12.5× IR collection objective (0.7 NA) and detected with an uncooled microbolometer focal plane array (480 × 480 pixels), yielding a field of view of 650 µm × 650 µm. In this configuration, the metasurface was placed in direct contact with a ceramic heater controlled by a programmable power supply, allowing temperature variation from 300 K to 500 K. All measurements were referred to by air.

### Data extraction

Peak resonance frequencies and Q-factors in Figs. 2 and 3 were obtained by fitting the experimentally measured transmittance spectra to the CIT formula (see SI Section II for details):

$${T}_{\mathrm{CIT}}={\left|1-\frac{i\left({{\rm{\omega }}}_{2}-{\rm{\omega }}\right){{\rm{\gamma }}}_{1}+i\left({{\rm{\omega }}}_{1}-{\rm{\omega }}\right){{\rm{\gamma }}}_{2}}{\left[i\left({{\rm{\omega }}}_{2}-{\rm{\omega }}\right)+{{\rm{\gamma }}}_{2}\right]\left[i\left({{\rm{\omega }}}_{1}-{\rm{\omega }}\right)+{{\rm{\gamma }}}_{1}\right]-{{\rm{\gamma }}}_{1}{{\rm{\gamma }}}_{2}-{\rm{Im}}{\left({\rm{\kappa }}\right)}^{2}}\right|}^{2}$$

Here, ω_1_ and ω_2_ denote the resonance frequencies of the two cavity modes, while γ_1_ and γ_2_ represent their corresponding total radiative decay rates and ${\rm{\kappa }}$ describes the direct cross-coupling between different modes.

The resonance frequency of CIT mode can be given as

$${{\rm{\omega }}}_{\mathrm{CIT}}=\frac{{{\rm{\omega }}}_{1}{{\rm{\gamma }}}_{2}+{{\rm{\omega }}}_{2}{{\rm{\gamma }}}_{1}}{{{\rm{\gamma }}}_{1}+{{\rm{\gamma }}}_{2}}$$

## Supplementary information

Supplementary Information for Dynamically Tunable Membrane Metasurfaces for Infrared Spectroscopy and Strong Light-Matter Interactions

## Data Availability

The data that support the findings of this study are available from the corresponding author upon reasonable request.
