# Supplementary material for: Dynamically tunable membrane metasurfaces for infrared spectroscopy and strong light-matter interactions
Source: Light Sci Appl. 2026 Jun 9;15:269. doi: 10.1038/s41377-026-02382-7 (PMC13249898; doi:10.1038/s41377-026-02382-7)
Supplement: Supplementary file 1 — Supplementary Information for Dynamically Tunable Membrane Metasurfaces for Infrared Spectroscopy and Strong Light-Matter Interactions [file 41377_2026_2382_MOESM1_ESM.docx]

Supplementary Information for

**Dynamically Tunable Membrane Metasurfaces for Infrared Spectroscopy and Strong Light-Matter Interactions**

Furkan Kuruoglu^1,2+^, Samir Rosas^1+^, Yihong Chen^3^, Brijesh Kumar^4^, Shenwei Yin^3^, Jin-Woo Cho^3^, David A. Czaplewski^5^, Yuri Kivshar^4^, Mikhail A. Kats^3^, Filiz Yesilkoy^1^*

*^1^Department of Biomedical Engineering, University of Wisconsin-Madison, Madison, Wisconsin 53706, USA*

*^2^Department of Physics, Faculty of Science, Istanbul University, Vezneciler, 34134, Istanbul, Turkey*

*^3^Department of Electrical and Computer Engineering, University of Wisconsin-Madison, Madison, Wisconsin 53706, USA*

*^4^Nonlinear Physics Center, Research School of Physics, Australian National University, Canberra ACT 2601, Australia*

*^5^Center for Nanoscale Materials, Argonne National Laboratory, Lemont, Illinois 60439, USA*

*Corresponding Author e-mail: filiz.yesilkoy@wisc.edu*

^+^equal contribution

Table of Contents

[I. Eigenmode Analysis 2](#_Toc222088818)

[A. Band-folding in diatomic unit cell: Origin of the BIC resonance 2](#_Toc222088819)

[B. Perturbation effect on Eigenmodes 2](#_Toc222088820)

[C. Impact of spectral proximity of SLM and q-BIC modes on the CIT line-shape 3](#_Toc222088821)

[II. Coupled-mode theory for coupling-induced transparency 4](#_Toc222088822)

[A. Two-mode, two-port system 4](#_Toc222088823)

[B. Steady-state solution and transmission 5](#_Toc222088824)

[C. Explicit two-mode, two-port expression 5](#_Toc222088825)

[i. $\kappa= 0$: Decoupled or orthogonal modes 6](#_Toc222088826)

[ii. $\kappa\neq0$: Coupled or non-orthogonal modes 6](#_Toc222088827)

[D. Validation of TCMT model 7](#_Toc222088828)

[III. Statistical Analysis and Residual Comparison of Fano and CIT Models 7](#_Toc222088829)

[IV. Geometric Parameter Sensitivity and Design Space of the CIT Resonance 9](#_Toc222088830)

[V. Phase Evolution of the Thermally Tuned Coupling-Induced Transparency Resonance 10](#_Toc222088831)

[VI. Simulated Radiative and Non-Radiative Loss Channels Under Thermal Tuning 11](#_Toc222088832)

[VII. Temperature-Dependent Electric Field Enhancement for Varying Tilt Angles (*θ = 2°– 8°*) 12](#_Toc222088833)

[VIII. Oxidation Regime and Its Influence on Resonance Stability 12](#_Toc222088834)

[IX. Thermal Cycling Stability of the Metasurface Resonance 14](#_Toc222088873)

[X. Resonantly Enhanced Absorption and Temperature-Dependent Transmission Attenuation 14](#_Toc222088874)

[XI. Spatial Uniformity of Resonance Position and Thermal Homogeneity 17](#_Toc222088875)

[XII. Temporal and Spatial Thermal Dynamics of the Suspended Silicon Membrane 18](#_Toc222088876)

[XIII. Temperature Dependence of the Intrinsic PMMA Vibrational Linewidth 19](#_Toc222088877)

[XIV. Thermal-Mechanical Stability of the Suspended Silicon Membrane 20](#_Toc222088878)

[XV. Quantitative Figure-of-Merit (FoM) Analysis for Tilt Angle Selection 20](#_Toc222088879)

[XVI. Practical Integration Pathway Toward Spectrometer-Free Operation 21](#_Toc222088880)

[XVII. References 22](#_Toc222088882)

# Eigenmode Analysis

## Band-folding in diatomic unit cell: Origin of the BIC resonance

Numerical simulations were carried out using the commercial software **COMSOL Multiphysics** to investigate the eigenmodes of the metasurface. The band structure along the $\Gamma-X$ direction of a single-atom (one elliptical hole in a unit cell) metasurface is shown in Figure S1(a), where the black circles indicate the surface lattice mode ($\omega_{SLM}$) and the high-Q guided mode ($\omega_{Q}$) before Brillouin zone folding (BZF). The filling color of the circles indicates each mode’s Q-factor as indicated by the colorbar. The first Brillouin zone (BZ) is outlined in Figure S1(c) indicating boundaries before and after BZF. Upon BZF, the bound states in the continuum (BIC) mode emerges at the $\Gamma$ point ($\omega_{Q}^{'}$), indicated with red circles in Figure S1(a). Figure S1(b) shows the calculated eigenvalues of a metasurface with a unit cell consisting of two elliptical dimers (diatomic unit cell). The $\omega_{Q}$ mode lies below the light line at the X point and is labeled $\omega_{Q}^{'}$ after folding, indicating that it behaves as a guided mode (GM) transversely confined by total internal reflection (TIR). The three bands, $\omega_{Q}^{'}=\omega_{BIC}$, $\omega_{SLM}$, and $\omega_{Q}$ that form upon BZF are shown in Figure S1(b).


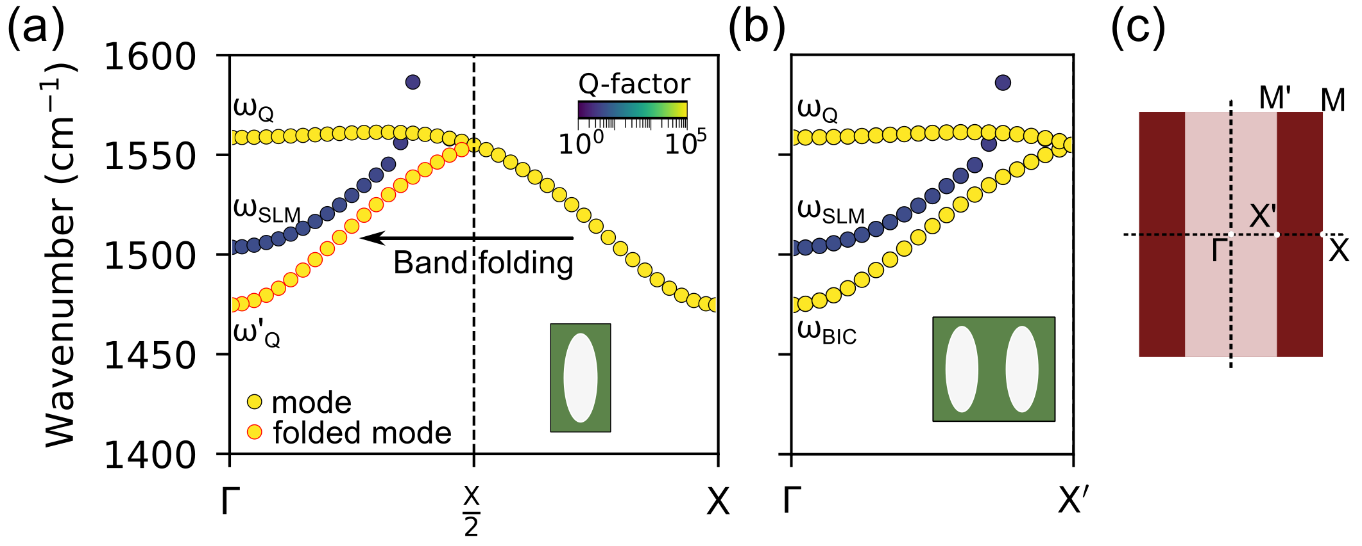


Figure S1: Brillouin zone folding-induced bound state in the continuum (BZF-BIC). a) Numerically calculated eigenmodes of a single-atom (elliptical hole) unit cell. The red circles highlight bands folded along $\text{X}\boldsymbol{/}\boldsymbol{2}$ b) Numerically calculated eigenmodes of a double-atom unit cell, showing excellent agreement with the folded band structure. c) Schematic of the Brillouin zone with high-symmetry points. (Only the relevant branches are plotted to maintain clarity.)

Introducing a periodic perturbation by doubling the periodicity of the photonic crystal (PhC) in the $x$ direction enables coupling of GMs to free space, thereby converting non-radiative dark modes into radiative resonances. This perturbation halves the Brillouin zone size, as shown by the pink box in the inset of Figure S1(c). As a result, the X point of the single ellipse unit cell of PhC is folded to the $\Gamma$ point, bringing the $\omega_{Q}^{'}$ mode into the radiation continuum, as clearly illustrated in Figure S1(a). The midpoint between $\Gamma$ and X in the unperturbed PhC becomes the X point of the perturbed structure by doubling the unit cell. To avoid ambiguity, this new point is denoted as $\text{X}^{\text{'}}=\frac{\text{X}}{2}$.

## Perturbation effect on Eigenmodes

BIC modes do not couple to the far field, therefore we introduced a perturbation via the diatomic unit cell design to bring the BIC mode into the BZF quasi-BIC (q-BIC) regime. We explored several types of perturbations:

1. Angle Perturbation in Ellipses, i.e. Rotating of Ellipses in opposite directions
2. Gap Perturbation, i.e. Changing Gap Between Ellipses
3. Eccentricity variation, i.e. by transforming ellipses into circles

All perturbations modify the spectral behaviour of the symmetry-protected BIC modes and the SLM mode in distinct ways (see Figure S2). However, only the **angle perturbation** provides a systematic and controllable tuning of the resonance linewidths and radiative coupling. The angle perturbation is detailed in the main text, here we show the alternative perturbations we investigated.


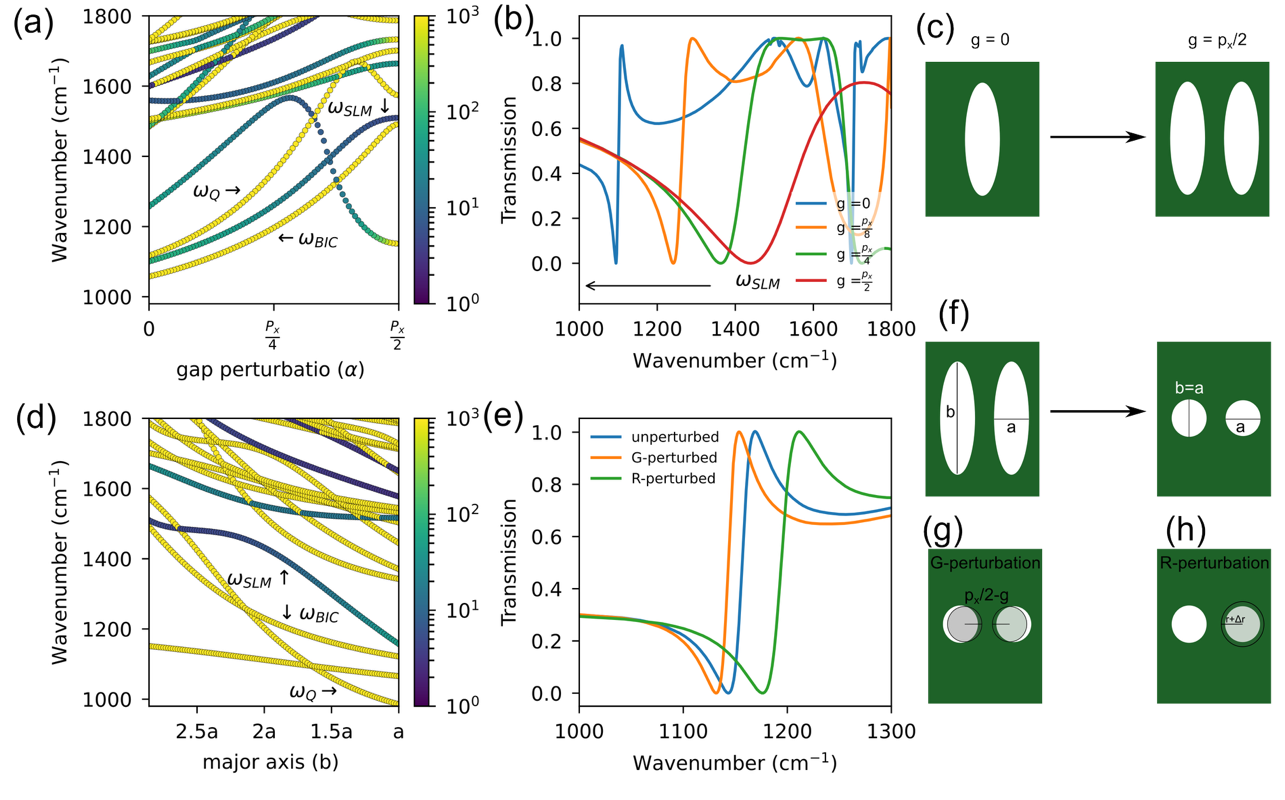


Figure S2: Different perturbation methods investigated to generate q-BIC modes: a) Eigenmode evolution as a function of the gap between two elliptical apertures within the diatomic unit cell. b) Corresponding simulated transmission spectra for different gap values ranging from 0 to p_x_/2, as illustrated schematically in c). d) Eigenmode evolution as a function of the major axis length of the elliptical apertures, gradually transforming the ellipses into circular apertures. e) Corresponding simulated transmission spectra for the major-axis perturbation shown in (f). c,f) Schematic illustrations of the unit cell geometries for gap and eccentricity perturbations, respectively. g,h) Schematic comparison between unperturbed and perturbed unit cells highlighting the symmetry properties of each configuration.

## Impact of spectral proximity of SLM and q-BIC modes on the CIT line-shape

We further investigated the impact of spectral proximity between the high-Q BZF-q-BIC ($\omega_{BIC}$) and the broad surface lattice modes ($\omega_{SLM}$) on the coupling-induced transparency (CIT) mode, which we measure in our metasurface transmission spectra. Figure S3 shows simulated spectra of a diatomic unit cell metasurface design with a constant tilting angle (*θ=6°*) at varying unit cell periods in x-direction (P_xnom_=3000 – 4200 nm). Since the SLM shifts slower than the q-BIC mode upon period change, the proximity between the modes decreases with decreasing period. Our analysis demonstrates that the spectral proximity of the SLM and BZF-q-BIC is important as it determines the Q-factor, asymmetry, as well as the transmission amplitude of the CIT mode.

Figure S3: Impact of spectral proximity between SLM and BZF-q-BIC modes on CIT mode. Numerically calculated dispersion and resonance characteristics illustrating the lattice period P_x_ is used to position the modes in spectral proximity. The adjusted P_xnom_ value (4200 nm) is employed to ensure coupling of the SLM and q-BIC modes within the target spectral region. As the P_xnom_ decreases, the spectral proximity decreases and the Q-factor and the transmission amplitude of the CIT mode decrease.

The band-folding and numerical simulation analyses provide a qualitative understanding of the CIT mode. In the following section, we develop a temporal coupled-mode theory (TCMT) model to quantitatively describe the coupling dynamics between the SLM and q-BIC modes and free-space channels.

# Coupled-mode theory for coupling-induced transparency

To quantitatively model the CIT that we observe in the transmission spectra of our membrane metasurface design, we employ the TCMT^1,2^ . In general, consider a resonator system with $n$ modes coupled to $m$ ports. The incoming waves from the ports can couple to the fundamental modes, as well as the coupled modes in the systems. The dynamics of such a system are described by

|  | $\frac{\text{d}}{\text{d}t}\boldsymbol{a}=\left( i\Omega-\Gamma+\kappa\right)\boldsymbol{a}+\mathcal{K}^{\mathcal{T}}s_{+}$ | (1) |
| --- | --- | --- |
|  | $s_{-}=\mathcal{C}s_{+}+\mathcal{D}\boldsymbol{a}$ | (2) |
|  | $\mathcal{D}^{\dagger}\mathcal{D}=2\Gamma$ | (3) |
|  | $\mathcal{K=D}$ | (4) |
|  | $\mathcal{C}\mathcal{D}^{*}\mathcal{=-D}$ | (5) |

Here, $\Omega$ and $\Gamma$ are $n\times n$ Hermitian matrices representing the resonant frequencies and decay rates, respectively. The vector $s_{+}$ contains the incoming wave amplitudes from the $m$ ports, coupled to the resonator through the $m\times n$ coupling matrix $\mathcal{K}$. The outgoing waves $s_{-}$ are generated via the coupling matrix $\mathcal{D}$. The off-diagonal $n\times n$ matrix $\kappa$ describes the direct cross-coupling between different modes while the matrix 𝒞 is the direct scattering matrix.

## Two-mode, two-port system

For our system, we consider two resonances coupled to two physical ports, with the matrices

|  | $\Omega=\left( \begin{matrix} \omega_{1} & 0 \\ 0 & \omega_{2} \end{matrix} \right),\quad\Gamma=\left( \begin{matrix} \gamma_{1} & 0 \\ 0 & \gamma_{2} \end{matrix} \right)$ | (6) | |
| --- | --- | --- | --- |
|  | $\mathcal{C=}\left( \begin{matrix} 0 & 1 \\ 1 & 0 \end{matrix} \right),\quad\mathcal{D=}\left( \begin{matrix} d_{11} & d_{12} \\ d_{21} & d_{22} \end{matrix} \right)$ | (7) | |
|  | $\kappa=\left( \begin{matrix} 0 & \kappa_{12} \\ \kappa_{21}^{*} & 0 \end{matrix} \right)$ | (8) |  |

Here, *ω_1_* and *ω_2_* denote the resonance frequencies of the two cavity modes, while γ_1_ and γ_2_ represent their corresponding total radiative decay rates. The coefficient d_ij_ denotes the complex coupling amplitude from the j-th resonant mode into the i-th port.

Imposing the relations from Eq. (3) and (5), and assuming mirror symmetry, we obtain^1^

|  | $\left\vert d_{11} \right\vert=\left\vert d_{21} \right\vert=\sqrt{\gamma_{1}}$ | (9) |
| --- | --- | --- |
|  | $\left\vert d_{22} \right\vert=\left\vert d_{12} \right\vert=\sqrt{\gamma_{2}}$ | (10) |

We now seek the steady-state solution of Eq. (1)-(5), which will allow us to express the transmission spectrum in terms of the modal parameters $\left( \omega_{1,2},\gamma_{1,2},\kappa_{12} \right)$.

## Steady-state solution and transmission

In the steady state, the resonator amplitudes satisfy

|  | $\frac{\text{d}}{\text{d}t}a=0$ | (11) |
| --- | --- | --- |

Thus, from Eq. (1), we have

|  | $0=\left( i\Omega-\Gamma+\kappa\right)a+\mathcal{K}^{\mathcal{T}}s_{+}$ | (12) |
| --- | --- | --- |
|  | $a=-\left( i\Omega-\Gamma+\kappa\right)^{-1}\mathcal{D}^{\mathcal{T}}s_{+}$ | (13) |

and the outgoing wave is

|  | $s_{-}=\left[ \mathcal{C}-\mathcal{D}\left( i\Omega-\Gamma+\kappa\right)^{-1}\mathcal{D}^{\mathcal{T}} \right]s_{+}$ | (14) |
| --- | --- | --- |

The transmission coefficient is therefore

|  | $t\left( \omega\right)=\frac{s_{-}}{s_{+}}$ | (15) |
| --- | --- | --- |
|  | $t\left( \omega\right)\mathcal{=C-D}\left( i\Omega-\Gamma+\kappa\right)^{-1}\mathcal{D}^{\mathcal{T}}$ | (16) |

## Explicit two-mode, two-port expression

Substituting the explicit two-mode matrices into Eq. (16), we obtain

|  | $t\left( \omega\right)=1-\frac{\left( i\left( \omega_{2}-\omega\right)+\gamma_{2} \right)\gamma_{1}+\left( i\left( \omega_{1}-\omega\right)+\gamma_{1} \right)\gamma_{2}-\left( \kappa+\kappa^{*} \right)\sqrt{\gamma_{1}\gamma_{2}}}{\left[ i\left( \omega_{2}-\omega\right)+\gamma_{2} \right]\left[ i\left( \omega_{1}-\omega\right)+\gamma_{1} \right]-\left\vert\kappa\right\vert^{2}}$ | (17) |
| --- | --- | --- |

Simplifying with $Re\left( \kappa\right)$ the transmission can be stated as:

|  | $T=\left\vert1-\frac{\left( i\left( \omega_{2}-\omega\right)+\gamma_{2} \right)\gamma_{1}+\left( i\left( \omega_{1}-\omega\right)+\gamma_{1} \right)\gamma_{2}-2Re\left( \kappa\right)\sqrt{\gamma_{1}\gamma_{2}}}{\left[ i\left( \omega_{2}-\omega\right)+\gamma_{2} \right]\left[ i\left( \omega_{1}-\omega\right)+\gamma_{1} \right]-\left\vert\kappa\right\vert^{2}} \right\vert^{2}$ | (18) |
| --- | --- | --- |

### $\boldsymbol{\kappa= 0}$: Decoupled or orthogonal modes

|  | $T=\left\vert1-\frac{\gamma_{1}}{i\left( \omega_{1}-\omega\right)+\gamma_{1}}-\frac{\gamma_{2}}{i\left( \omega_{2}-\omega\right)+\gamma_{2}} \right\vert^{2}$ | (19) |
| --- | --- | --- |

### $\boldsymbol{\kappa\neq}\boldsymbol{0}$: Coupled or non-orthogonal modes

CIT occurs when $T\left( \omega\right)=1$, i.e.

|  | $\left( i\left( \omega_{2}-\omega\right)+\gamma_{2} \right)\gamma_{1}+\left( i\left( \omega_{1}-\omega\right)+\gamma_{1} \right)\gamma_{2}-2Re\left( \kappa\right)\sqrt{\gamma_{1}\gamma_{2}}=0$ | (20) |
| --- | --- | --- |

|  | $\Rightarrow2Re\left( \kappa\right)\sqrt{\gamma_{1}\gamma_{2}}=2\gamma_{1}\gamma_{2}\quad\Rightarrow\quad\boxed{\mathrm{Re}\left( \kappa\right)=\sqrt{\gamma_{1}\gamma_{2}}}$ | (21) |
| --- | --- | --- |
|  | $\left( \omega_{2}-\omega\right)\gamma_{1}+\left( \omega_{1}-\omega\right)\gamma_{2}=0\quad\Rightarrow\quad\boxed{\omega_{\text{CIT}}=\frac{\omega_{1}\gamma_{2}+\omega_{2}\gamma_{1}}{\gamma_{1}+\gamma_{2}}}$ | (22) |

The transmission at the CIT condition then becomes:

|  | $\boxed{T_{\text{CIT}}=\left\vert1-\frac{i\left( \omega_{2}-\omega\right)\gamma_{1}+i\left( \omega_{1}-\omega\right)\gamma_{2}}{\left[ i\left( \omega_{2}-\omega\right)+\gamma_{2} \right]\left[ i\left( \omega_{1}-\omega\right)+\gamma_{1} \right]-\gamma_{1}\gamma_{2}-\mathrm{Im}\left( \kappa\right)^{2}} \right\vert^{2}}$ | (23) |
| --- | --- | --- |

**Figure S4:** **Validation of TCMT model on the CIT mode supported by our metasurface design:** We fitted our numerically calculated transmission spectra (grey dots) for different tilting angles (*θ* = 1°,2°,8°,15°) to Eq. (23) to verify TCMT model on the CIT mode. We chose $\omega_{1,2} \gamma_{1,2}$ from eigenmode solution and fitted for coupling parameter $\kappa$. In all panels, the grey circles show the simulated spectra and the blue solid lines show the fitted data. Black dashed lines show the spectral peak position ($\omega_{CIT}$) of the CIT mode, calculated using Eq.(22).

## Validation of TCMT model

To validate that the TCMT model explains the CIT mode supported by our metasurface design, we take $\omega_{1}$, $\omega_{2}$, $\gamma_{1}$, and $\gamma_{2}$ from Eigenmode analysis and insert these values in Eq. 23 using the curve-fit module from SciPy. We repeat this process for different tilting angles (*θ* = 1°,2°,8°,15°) of the diatomic unit cell design, which reveals the coupling constant $\kappa$ for each design as indicated in **Figure S4** (a-d). In the curve fitting process, we apply the CIT condition by setting $R\left( \kappa\right)=\sqrt{\gamma_{1}\gamma_{2}}$. We also calculated the wavenumber of the CIT mode using Eq.(22), which matches the transmission peak in simulated spectra as indicated with the black dashed lines in each panel.

# Statistical Analysis and Residual Comparison of Fano and CIT Models

Table S1: Experimentally extracted Q-factors and coefficients of determination (R²) obtained from Fano and CIT fits to measured transmission spectra collected at 300 K and 700 K from metasurfaces with tilt angles *θ = 2°–8°*.

|  | 300 K | | | | 700 K | | | |
| --- | --- | --- | --- | --- | --- | --- | --- | --- |
| Tilt Angle | Q-Factor by Fano Fit | R^2^ | Q-Factor by CIT Fit | R^2^ | Q-Factor by Fano Fit | R^2^ | Q-Factor by CIT Fit | R^2^ |
|  |  | (Fano) |  | (CIT) |  | (Fano) |  | (CIT) |
| 2 | 178.965 | 0.675 | 230.000 | 0.949 | 53.141 | 0.849 | 158.612 | 0.867 |
| 3 | 140.969 | 0.899 | 161.612 | 0.971 | 152.239 | 0.799 | 126.996 | 0.856 |
| 4 | 147.518 | 0.975 | 143.659 | 0.990 | 119.819 | 0.897 | 127.096 | 0.945 |
| 5 | 97.394 | 0.980 | 129.096 | 0.998 | 110.226 | 0.920 | 115.272 | 0.983 |
| 6 | 79.673 | 0.982 | 107.501 | 0.999 | 101.751 | 0.954 | 97.379 | 0.997 |
| 7 | 82.154 | 0.987 | 91.429 | 0.998 | 86.285 | 0.914 | 89.860 | 0.995 |
| 8 | 87.887 | 0.978 | 90.646 | 0.998 | 83.456 | 0.945 | 95.848 | 0.995 |

We extracted the resonance frequencies and Q-factors by fitting the measured transmission spectra to Fano and CIT models for statistical comparison. For each spectrum measured from metasurfaces with varying tilting angles (*θ =* 2°–8°) across the full temperature range (300 – 700 K), both models were applied using identical fitting windows. The fitting residuals were evaluated in terms of mean absolute residual. The resulting residual statistics are summarized in Table S1, and representative residual plots for selected temperatures are shown in Figure S5. The results show that the CIT model provides consistently lower residuals than the Fano model, verifying that our membrane metasurface supports a CIT resonance mode.

Figure S5: Representative experimental transmission spectrum fitted using Fano and CIT models (top), and their corresponding residuals (bottom). The CIT model exhibits reduced residual amplitude and improved agreement near the transparency window.

# Geometric Parameter Sensitivity and Design Space of the CIT Resonance

Figure S6: Simulation of the CIT resonance mode as a function of geometric design parameters (*θ*, *P_x_, P_y_, h, a* and *b*). All parameters were fixed at *θ* = 6°, *h* =1000 nm, *P_x_* = 4000 nm, *P_y_* = 4400 nm, *a* = 1400 nm and *b* = 4000 nm, while sweeping only one parameter at a time. a) Parametric variation of *θ* from 0° to 12°. b), Parametric variation of *h* from 500 nm to 1500 nm. c), Parametric variation of *P_x_* from 3700 nm to 4400 nm. d), Parametric variation of *P_y_* from 4200 nm to 4700 nm. e), Parametric variation of *a* from 1200 nm to 1600 nm. f), Parametric variation of *b* from 3500 nm to 4100 nm. All units in (nm).

Parametric simulations performed using CST map the sensitivity of the CIT resonance to individual geometric degrees of freedom while preserving all other structural parameters. The results show that the resonance position and linewidth respond differently to symmetry-breaking parameters (tilt angle) and symmetry-preserving parameters (lattice constants and aperture dimensions). Variation of the tilt angle primarily controls the radiative coupling strength and linewidth of the resonance, whereas changes in membrane thickness, lattice periodicities, and aperture dimensions predominantly shift the resonance frequency while maintaining the overall transmission contrast in the considered range. This systematic parameter sweep defines the accessible design space of the metasurface and provides practical guidance for selecting geometric parameters to target specific spectral regions while preserving high-Q CIT behavior.

# Phase Evolution of the Thermally Tuned Coupling-Induced Transparency Resonance

Figure S7: Phase properties of the CIT mode. a,b) Simulated phase maps of the metasurface transmission as a function of symmetry breaking double rod tilt angle in a unit cell and wavenumber at 300 K (a) and 700 K (b), illustrating the evolution of the phase dispersion associated with the thermally tuned CIT resonance. c) Corresponding phase (solid lines) and transmittance (open symbols) spectra at *θ =* 6°, showing the characteristic phase jump corresponding to two coupled modes and CIT mode transmission peak at both temperatures.

Figure S7 presents the simulated phase response of the metasurface transmission spectra at 300 K and 700 K and provides additional confirmation on the thermal tunability of the CIT mode. The simultaneous observation of a sharp transmission peak and a rapid phase jump at the same spectral position is a defining signature of a resonance mode. The preservation of this phase behavior at elevated temperature (700 K) indicates that thermal tuning modifies the resonance frequency without altering the fundamental interference mechanism governing the metasurface spectral response.

# Simulated Radiative and Non-Radiative Loss Channels Under Thermal Tuning

Figure S8 presents a simulation-based decomposition of the loss channels contributing to the temperature-dependent evolution of the resonance Q-factor discussed in the main text (Figure 2g). The results indicate that the reduction of the Q-factor at elevated temperatures is dominated by an increase in non-radiative damping, while the radiative loss channel remains comparatively insensitive to temperature. This behavior reflects the temperature dependence of the complex refractive index of crystalline silicon in the mid-infrared and confirms that thermal tuning primarily affects material absorption rather than the radiative coupling strength of the q-BIC-CIT resonance.

The loss rates were obtained with the following Fano fit from^3^

| $T=\left\vert ie^{i\phi}t_{0}+\frac{\Gamma_{R}}{\Gamma_{R}+ \Gamma_{NR}+i(\lambda-\lambda_{res})} \right\vert^{2}$ | (24) |
| --- | --- |
| Where ${ie}^{i\phi}t_{0}$describes the background and the shape of the resonance. The Q-factor can then be calculated as:  $Q=\frac{\lambda_{res}}{2(\Gamma_{R}+ \Gamma_{NR})}$ | (25) |

Where $\lambda_{res}$, $\Gamma_{R}$, and $\Gamma_{NR}$are the resonance wavelength, radiative, and absorptive loss rates, respectively.

Figure S8: Simulated radiative damping rates *Γ_rad_* (left panel) and non-radiative damping rates *Γ_non-rad_* (right panel) as a function of temperature for metasurfaces with tilt angles *θ =* 2°, 4°, 6°, and 8°. The simulations show a slight temperature dependence of *Γ_rad_*, whereas *Γ_non-rad_* increases markedly at elevated temperatures (T>550 K), indicating enhanced absorption-related losses in crystalline silicon.

# Temperature-Dependent Electric Field Enhancement for Varying Tilt Angles (*θ =* 2°– 8°)

Table S2: Temperature-dependent E-field enhancement for metasurfaces with different tilt angles, a geometric parameter that impacts the radiative mode coupling.

| Tilt Angle (degrees) | E_enh_ (300 K) | E_enh_ (700 K) | \|∆E_enh_\| | Ratio $\left( \frac{E_{\mathrm{enh}}300 K}{E_{\mathrm{enh}}700 K} \right)$ | Change (%) |
| --- | --- | --- | --- | --- | --- |
| 2 | 44.17 | 32.77 | 11.4 | 0.741 | 25.8 |
| 4 | 24.45 | 23.05 | 1.4 | 0.942 | 5.7 |
| 6 | 16.80 | 16.58 | 0.22 | 0.986 | 1.3 |
| 8 | 13.66 | 13.7 | 0.04 | 1.003 | 0 |

# Oxidation Regime and Its Influence on Resonance Stability

Within the temperature range investigated in this work (≤700 K), silicon oxidation remains in a self-limiting native-oxide regime rather than following classical Deal–Grove kinetics ^4^. While a temperature induced surface oxidation in our Si metasurface is not expected, to answer a reviewer’s concern, we evaluated the potential impact of such oxide formation on resonance behavior. Electromagnetic simulations were performed on silicon membrane metasurface with a surface oxide thickness between 0 to 10 nm. Even under this extreme scenario, the resulting effect of a thin silicon oxide film on the resonance is limited to a ~ 3 cm^-1^ shift and no measurable effects were observed on the Q-factor.


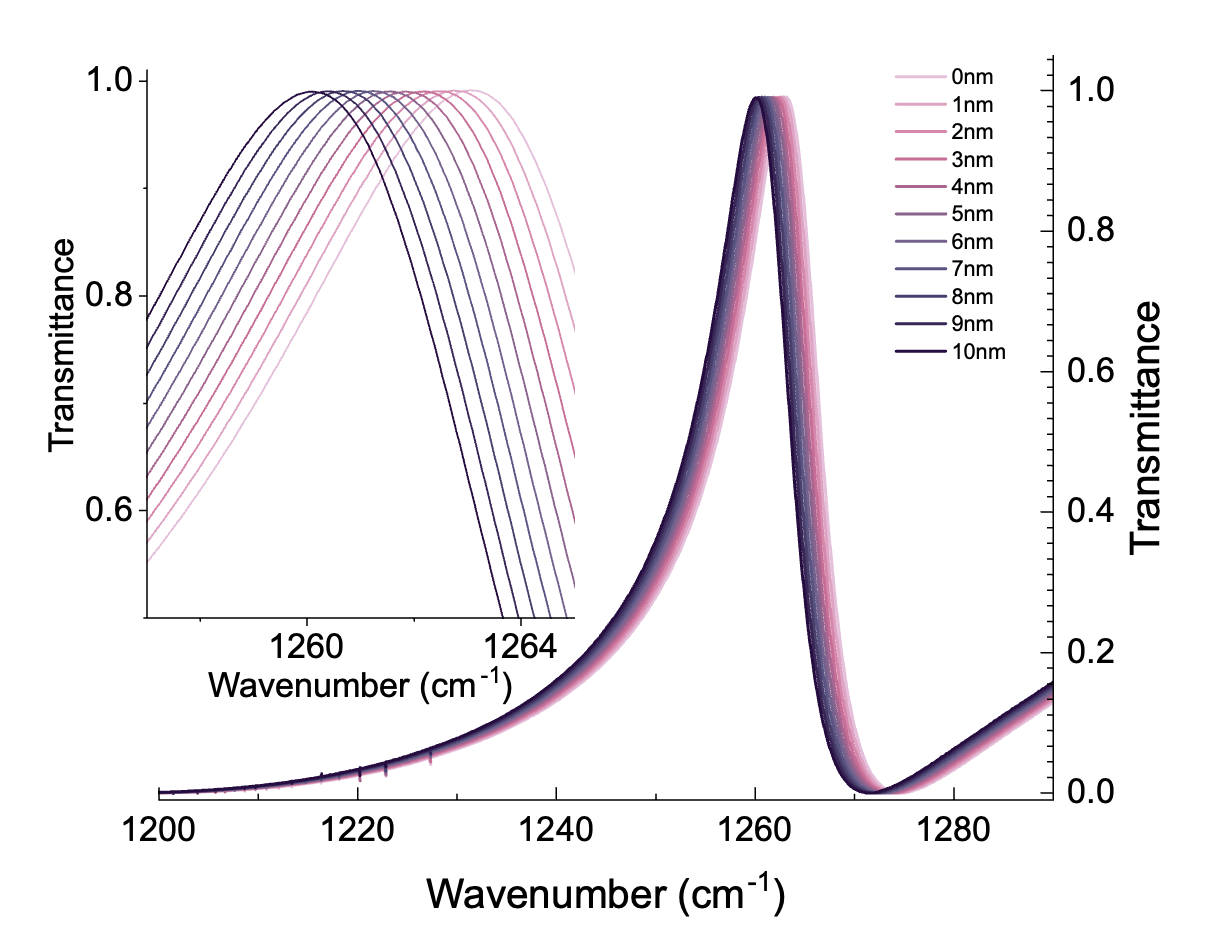


Figure S9 Simulated transmittance spectra of the membrane metasurface considering an extreme scenario corresponding to maximal native-oxide–like growth expected below 500 K. The comparison demonstrates that even under this upper-bound oxidation condition, the resonance shift remains limited to approximately 3 cm^-1^. This result confirms that self-limiting native oxide formation in this temperature regime does not qualitatively affect the resonance behavior or device operation.

# Thermal Cycling Stability of the Metasurface Resonance

Figure S10: a) Experimentally measured transmission spectra of the same metasurface at 300 K, 500 K, and 700 K, illustrating the thermal tuning of the resonance. b) Extracted peak resonance wavenumber as a function of thermal cycle number during repeated heating and cooling between 300 K and 700 K. Data points show both heat-up and cool-down branches at each temperature. The negligible drift and close overlap between heating and cooling traces confirm minimal hysteresis and stable optical performance under repeated thermal cycling.

Repeated thermal cycling between 300 K and 700 K results in reversible resonance tuning with negligible spectral drift. The close overlap in resonance peak wavenumbers measured during heating and cooling cycles indicates minimal thermal hysteresis and stable resonance behavior under repeated thermal cycling. These measurements confirm that thermal modulation does not induce permanent structural or optical degradation of the metasurface and demonstrate the robustness of the silicon membrane platform for repeated dynamic tuning.

# Resonantly Enhanced Absorption and Temperature-Dependent Transmission Attenuation

The standard thermo-optic picture (temperature-dependent real refractive index, ∆n) correctly predicts the spectral redshift of the resonance, but it does not explain the experimentally observed strong reduction in transmission magnitude. In resonant photonic structures, absorption is not purely a “bulk” material property; instead, the absorbed electromagnetic power is field-weighted and can therefore be strongly amplified by resonant field localization. For nonmagnetic materials, the absorbed power can be written in a compact form as

| $P_{\text{abs}}=\frac{1}{2} \omega\text{Im}\left( \varepsilon\right) \left\vert E \right\vert^{2}$ | (26) |
| --- | --- |

where *P_abs_* is the time-averaged absorbed optical power, 𝜔 is the frequency of light, 𝜀 is the complex relative permittivity of the absorbing medium, *Im(𝜀*) is its imaginary part (representing intrinsic material absorption), and E is the local electric-field amplitude inside the absorbing region^5^.

An equivalent and widely used formulation expresses the absorbed electromagnetic power through the volume integral of the current–field product,

| $P=\frac{1}{2} \text{Re}\left\{ \int_{V} \boldsymbol{J}\left( \boldsymbol{r} \right)\cdot\boldsymbol{E}\left( \boldsymbol{r} \right) dV \right\}$ | (27) |
| --- | --- |

where the current density *J=𝜎E* and *𝜎=𝜀_0_𝜔Im(𝜀)* ^6^.  This representation highlights the central point: stronger electric fields inside the resonator directly increase the absorbed power, even if the intrinsic material loss is modest.

This field-weighted loss amplification mechanism is closely analogous to surface-enhanced infrared absorption (SEIRA/SEIRAS), where the observed absorption intensity by a molecule in the close vicinity of a resonator scales with the square of the local electric field at the surface. In SEIRAS, the absorption can be written as

| $A\propto\left\vert\frac{\partial\mu}{\partial Q}\cdot E \right\vert^{2}=\left\vert\frac{\partial\mu}{\partial Q} \right\vert^{2}\left\vert E \right\vert^{2}\cos^{2} \theta$ | (28) |
| --- | --- |

where A is the infrared absorption intensity, μ is the molecular dipole moment, 𝑄 is the vibrational normal coordinate, ∂μ/∂𝑄 is the dipole-moment derivative associated with the vibrational mode, *E* is the local electric field driving the vibration, and *θ* is the angle between the dipole derivative vector and the local electric-field direction. This derivation emphasizes that enhancement arises because the local *|E|^2^* near the surface can significantly exceed the incident field intensity^7^. In our membrane metasurface, the high-Q resonance similarly concentrates the electric field inside the silicon membrane. Therefore, any temperature-induced increase in the silicon loss (i.e., increase in *Im(𝜀)* or equivalently k) is weighted by the resonantly enhanced *|E|^2^* and manifests as an amplified attenuation in transmittance.

This is also consistent with the general understanding of quasi-bound states in the continuum (quasi-BICs), where strong confinement and field localization boost dissipative losses because the power-loss density scales with *Im(𝜀)|E|^2^* ^8^. Moreover, resonant absorption and thermal/photothermal metasurface concepts consistently rely on the fact that localized electromagnetic energy can amplify effective absorption and dissipation in the active material^9^.

Figure S11: Simulated transmission spectra of the metasurface resonance mode (*θ =* 6°) as a function of temperature from 300 K to 700 K after applying a temperature-dependent artificial extinction coefficient to account for resonant near-field enhanced absorption inside the silicon membrane. The simulations reproduce the experimentally observed progressive resonance redshift and transmission attenuation. Inset: Temperature dependence of the artificial extinction coefficient (left axis) and the corresponding normalized resonant electric-field intensity enhancement |E|/|E_0_|^2^ (right axis).

To directly verify that the observed attenuation is governed by field-weighted absorption (rather than a change in radiative coupling or resonance disappearance), we introduce an effective extinction coefficient in the simulations to account for the resonantly enhanced near-field intensity within the silicon membrane, analogous to the electromagnetic enhancement mechanism in SEIRAS where absorption scales with the local |*E|^2^* ^7^. Temperature-dependent transmission attenuation is reproduced in the simulations when an effective extinction coefficient is introduced, adjusting for resonant near-field absorption enhancement inside the silicon membrane. The extracted extinction coefficient follows the same temperature trend as the normalized local E-field intensity enhancement, indicating that the effective absorption scales with resonantly enhanced light–matter interaction rather than changes in radiative coupling. This correlation supports the interpretation that the observed reduction in transmittance at elevated temperatures originates from field-weighted material absorption in crystalline silicon while preserving the underlying CIT resonance mechanism. This behavior is consistent with the general absorbed-power formalism $P_{\text{abs}}\propto\omega\text{Im}\left( \varepsilon\right) \left| E \right|^{2}$, which explicitly predicts that increased intrinsic loss becomes disproportionately visible under strong resonant field confinement^5,6,8^.

# Spatial Uniformity of Resonance Position and Thermal Homogeneity

Figure S12: a) Spatial maps of the experimentally extracted peak resonance wavenumbers across a single metasurface area of 400 µm x 400 µm at 300 K and 500 K, showing a standard deviation of σ = 8.00 cm^-1^ at 300 K and σ = 10.55 cm^-1^ at 500 K over the center dashed area. b) Corresponding spatial distribution of the resonance wavenumber shift across the same area. The magnitude of the spatial variations in wavenumber shift is comparable to the minimum wavenumber step of the optical measurement system (2 cm^-1^), confirming that the membrane exhibits sufficient thermal uniformity and that temperature nonuniformity does not measurably distort the transmittance spectra shown in Figure 3d. c) Distribution of average peak wavenumbers and associated colormaps for the metasurface arrays fabricated on two different chips, which were used to measure poly(methyl methacrylate) (PMMA) and polystyrene (PS), illustrating almost uniform temperature-induced resonance shifts across the metasurface arrays.

Spatial mapping of the peak resonance wavenumber both within a single metasurface area (Figure S11a,b) and across a metasurface array demonstrates a high thermal and spectral uniformity across the membrane area (3 mm x 3 mm). The magnitude of spatial variations in resonance position remains comparable to or smaller than the minimum wavenumber step of the measurement system (2 cm^-1^), indicating that temperature nonuniformity does not measurably distort the transmittance spectra presented in the main text (Figure 3d). The linear and consistent resonance shift observed across the angle-dependent membrane arrays shown in Figure 3b further confirms homogeneous temperature conditions during thermal tuning. Together, these results verify that the extracted spectral features arise from controlled thermo-optical modulation and fabrication-induced inhomogeneities rather than from spatial temperature gradients.

# Temporal and Spatial Thermal Dynamics of the Suspended Silicon Membrane

Figure S13: a) Time-resolved spatial temperature maps of the suspended silicon membrane at selected time delays (0, 10, 30, and 50 ms) during the heating process, obtained from transient thermal simulations. The bottom schematic illustrates the cross-sectional geometry used in the simulation model. b) Temporal evolution of the membrane surface temperature, illustrating the heat-up and cool-down dynamics between 300 K and 700 K and defining the effective thermal rise and recovery times. c) Spatial temperature profiles across the membrane width at different delay times, showing the evolution of temperature change during the transient heating process and becoming uniform at steady state. d) Simulated in-plane temperature profiles at t = 10 ms for suspended Si membranes with different lateral dimensions (1 × 1 mm, 3 × 3 mm, and 5 × 5 mm) during heating from 300 K to 700 K. The 1 × 1 mm membrane (blue) has reached the steady state at 10 ms and maintains near-uniform temperature across the active region, whereas larger membranes are still at the transient phase and exhibit increasing in-plane gradients. e) Transient thermal response of the suspended Si membranes with different lateral dimensions (1 × 1 mm, 3 × 3 mm, and 5 × 5 mm) during heating from 300 K to 700 K. While the smallest membrane reaches steady state temperature within ~6 ms under the applied heating conditions, it takes ~100 ms for the 5 × 5 mm^2^ sized membrane at the same heating conditions. The results confirm that the suspended membranes reach thermal equilibrium on millisecond timescales depending on their dimensions, supporting real-time thermal tuning during spectroscopic measurements.

Figure S13 presents transient thermal simulations of the free-standing silicon membrane and provides insight into the temporal and spatial thermal response of the metasurface platform. Owing to the low thermal mass and high in-plane thermal conductivity of the membrane, thermal equilibration occurs at millisecond timescales, with minimal spatial temperature gradients once the steady state is reached across the membrane region. The rapid and uniform thermal response confirms that the membrane itself does not limit the speed or homogeneity of thermal tuning, supporting its suitability for dynamic modulation of high-Q metasurface resonances.

# Temperature Dependence of PMMA Absorbance Bands

Figure S14 Transmittance spectra measured between 300 K and 500 K, highlighting the ∼1700–1760 cm^-1^ region used for peak analysis (inset). At 300 K, the fitted peak position is x₀ = 1730.41 cm^-1^ with a FWHM of 30.03 cm^-1^. At 500 K, the peak appears at x₀ = 1731.33 cm^-1^ with FWHM = 30.93 cm^-1^.

To separate intrinsic molecular effects from coupled photonic contributions, temperature-dependent transmission spectra of a CaF_2_-supported PMMA film (700 nm thickness) were measured over the 300 – 500 K range. Statistical analysis of repeated spectral fits indicates that the intrinsic PMMA bandwidth does not change significantly across the measured temperature range, while only a modest change in the peak position and transmission level was observed with increasing temperature. This result supports that the systematic linewidth broadening of the upper and lower polariton branches shown in Figure 5d is likely dominated by the temperature-dependent loss and decoherence mechanisms associated with the photonic resonance in the coupled system, including increased non-radiative damping in silicon and reduced coherence of the high-Q resonance at elevated temperatures.

# Thermal-Mechanical Stability of the Suspended Silicon Membrane

Figure S15: a–c) Optical views of the membrane during a rapid temperature ramp from 24 °C to 427 °C at 50 °C min^-1^ using an electrical ceramic heater (Linkam stage). The suspended membrane remains flat and mechanically stable without observable bowing, buckling, or deformation at room temperature a), intermediate temperature (224 °C) b), and at the maximum operating temperature (427 °C) c). Insets show a zoomed-in view of the metasurface area, confirming structural integrity under thermal load.

# Quantitative Figure-of-Merit (FoM) Analysis for Tilt Angle Selection


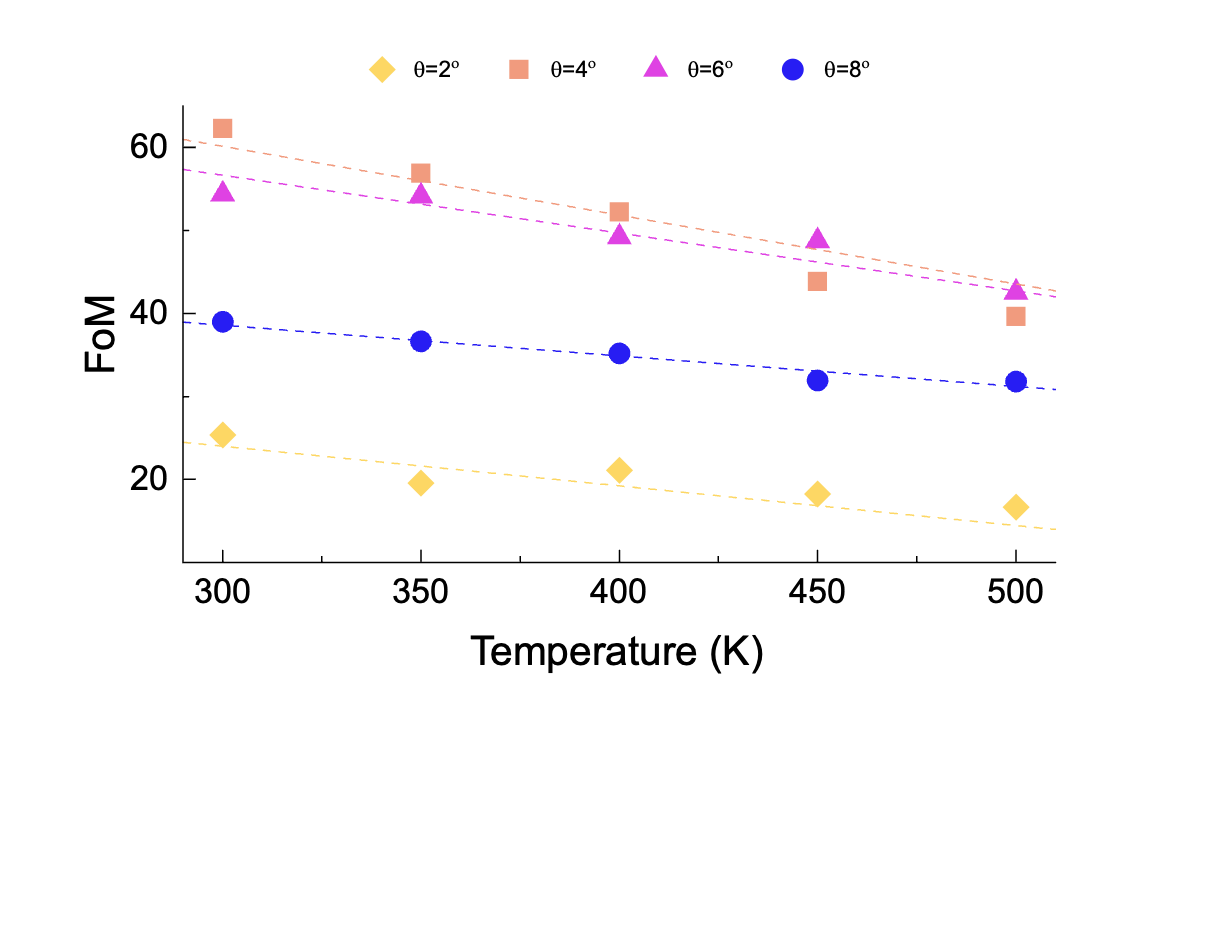


Figure S16 Temperature-dependent figure of merit (FoM) defined as $\text{FoM}\boldsymbol{=Q}\boldsymbol{\times}\boldsymbol{I}_{\text{peak}}$, where Q is the extracted resonance quality factor and I_peak_ is the transmission peak intensity, for metasurfaces with tilt angles *θ* = 2°, 4°, 6°, and 8° over the 300 – 500 K range. The metric combines spectral selectivity and measurable transmission contrast to evaluate practical sensing performance. Among the investigated geometries, *θ* = 6° maintains a high FoM across the entire temperature range while exhibiting stable behavior with increasing temperature.

To quantitatively assess the trade-off between spectral selectivity and measurable transmission contrast across different rod tilting angles, a figure of merit (FoM) is defined as:

| $\text{FoM}\boldsymbol{=Q}\boldsymbol{\times}\boldsymbol{I}_{\text{peak}}$ | (29) |
| --- | --- |

where Q is the extracted resonance quality factor and I_peak_ is the transmission peak intensity^10,11^.

This combined metric reflects both spectral sharpness and detectable signal strength, both of which are required for practical sensing operations.

Temperature-dependent FoM values for *θ* = 2°, 4°, 6°, and 8° over the 300 – 500 K range are presented in Figure S16. While smaller tilt angles yield higher Q at room temperature, their lower transmission intensity and stronger thermal sensitivity reduce the overall FoM. Larger angles provide more stable transmission but significantly reduced Q-factor.

*θ* = 6° consistently exhibits the highest FoM across the investigated temperature range while maintaining stable behavior with increasing temperature, confirming it as a balanced and thermally robust operating point.

# Practical Integration Pathway Toward Spectrometer-Free Operation

We propose a practical integration pathway using the same principles as a wide-field imaging setup, where a broadband light source evenly illuminates the metasurface array and transmitted light is imaged by a camera. To ensure uniform illumination, principles from established Kohler illumination can be used together with a 4-f imaging optics. A key aspect of our approach is that each metasurface array will be characterized a priori, recording their spatially resolved spectral properties. More specifically, the resonance peak wavenumbers and transmittance as a function of temperature will be mapped during manufacturing and saved as a device-specific calibration table. During analyte measurement operation, the system records two sets of images, one before (I₀_x_(T)) and one after (Iₓ(T)) analyte insertion. Then, using the metasurface-chip specific calibration table, the intensity recordings are reconstructed as absorbance spectra of the measured analyte. In summary, we propose a device where spectral information is encoded into a temperature-dependent transmission response of the metasurface array rather than using dispersive or interferometric optics.

**Figure S17 *For a spectrometer-less integrated mid-IR spectrometer approach,*** *a* broadband mid-infrared source *uniformly* illuminates *the* metasurface array with distinct resonance positions. The transmitted intensity from *metasurface* array is measured using an IR detector. Since the resonance *wavenumbers* *of the metasurface array are pre-recorded* as a function of temperature, the normalized intensity signal, −log(Iₓ(T)/I₀_x_(T)), *where* Iₓ(T) *and* I₀_x_(T) *are with and without analyte, respectively,* can be *correlated to analyte* molecul*e’s* absorption *spectrum*. By combining the temperature-dependent intensity responses from different *metasurfaces in the array*, the absorbance spectrum of *analyte molecules, e.g.,* PMMA*,* can be retrieved, demonstrating the principle of spectrometer-free chemical sensing.

# References

1 Suh W, Wang Z, Fan S. Temporal Coupled-Mode Theory and the Presence of Non-Orthogonal Modes in Lossless Multimode Cavities. *IEEE J Quantum Electron* 2004; **40**: 1511–1518.

2 Maksimov DN, Pankin PS, Kim D-W, Song M, Peng C, Bogdanov AA. Temporal coupled mode theory: From bound states in the continuum to uniguided resonances. *Phys Rev B* 2025; **112**: 235303.

3 Fan S, Suh W, Joannopoulos JD. Temporal coupled-mode theory for the Fano resonance in optical resonators. *J Opt Soc Am A, Opt, image Sci, Vis* 2003; **20**: 569–72.

4 Enta Y, Mun BS, Rossi M, Ross PN, Hussain Z, Fadley CS *et al.* Real-time observation of the dry oxidation of the Si(100) surface with ambient pressure x-ray photoelectron spectroscopy. *Appl Phys Lett* 2008; **92**: 012110.

5 Ma C, Yan J, Huang Y, Wang C, Yang G. The optical duality of tellurium nanoparticles for broadband solar energy harvesting and efficient photothermal conversion. *Sci Adv* 2018; **4**: eaas9894.

6 Zograf GP, Petrov MI, Zuev DA, Dmitriev PA, Milichko VA, Makarov SV *et al.* Resonant Nonplasmonic Nanoparticles for Efficient Temperature-Feedback Optical Heating. *Nano Lett* 2017; **17**: 2945–2952.

7 Osawa M. Surface-Enhanced Infrared Absorption. In: Kawata"] ["Satoshi (ed). *Near-Field Optics and Surface Plasmon Polaritons*. Springer: Berlin, Heidelberg, 2001, pp 163–187.

8 Wu J, Sun Y, Wu F, Wu B, Wu X. Tunable high-quality-factor absorption in a graphene monolayer based on quasi-bound states in the continuum. *Beilstein J Nanotechnol* 2022; **13**: 675–681.

9 Ijaz S, Kang D, Rana AS, Kim J, Chani MTS, Zubair M *et al.* Metasurface Absorber–Emitter Pair-Integrated High-Efficiency Thermophotovoltaic System. *ACS Photonics* 2025; **12**: 3829–3839.

10 Yang H, Chen Y, Liu M, Xiao G, Luo Y, Liu H *et al.* High Q-Factor Hybrid Metamaterial Waveguide Multi-Fano Resonance Sensor in the Visible Wavelength Range. *Nanomaterials* 2021; **11**: 1583.

11 Lee E, Seo IC, Lim SC, Jeong HY, Jun YC. Active switching and tuning of sharp Fano resonances in the mid-infrared spectral region. *Opt Express* 2016; **24**: 25684.
